# The role of *FMR1* mRNA structure on the efficiency of non-canonical translation of toxic polyglycine protein

**DOI:** 10.1093/nar/gkag569

**Published:** 2026-06-16

**Authors:** Daria Niewiadomska, Agnieszka Piasecka, Anna Baud, Izabela Broniarek, Krzysztof Sobczak

**Affiliations:** Department of Gene Expression, Institute of Molecular Biology and Biotechnology, Adam Mickiewicz University, Uniwersytetu Poznanskiego 6, 61-614 Poznan, Poland; Center for Development of Gene Therapies, Center for Advanced Technologies, Adam Mickiewicz University, Uniwersytetu Poznanskiego 10, 61-614 Poznan, Poland; Department of Gene Expression, Institute of Molecular Biology and Biotechnology, Adam Mickiewicz University, Uniwersytetu Poznanskiego 6, 61-614 Poznan, Poland; Department of Gene Expression, Institute of Molecular Biology and Biotechnology, Adam Mickiewicz University, Uniwersytetu Poznanskiego 6, 61-614 Poznan, Poland; Center for Development of Gene Therapies, Center for Advanced Technologies, Adam Mickiewicz University, Uniwersytetu Poznanskiego 10, 61-614 Poznan, Poland; Department of Gene Expression, Institute of Molecular Biology and Biotechnology, Adam Mickiewicz University, Uniwersytetu Poznanskiego 6, 61-614 Poznan, Poland; Department of Gene Expression, Institute of Molecular Biology and Biotechnology, Adam Mickiewicz University, Uniwersytetu Poznanskiego 6, 61-614 Poznan, Poland; Center for Development of Gene Therapies, Center for Advanced Technologies, Adam Mickiewicz University, Uniwersytetu Poznanskiego 10, 61-614 Poznan, Poland

## Abstract

Repeat-associated non-AUG (RAN) translation of mutant *FMR1* messenger RNA (mRNA) containing CGG repeat expansions results in the production of a toxic polyglycine protein (FMRpolyG), which contributes to fragile X premutation-associated conditions (FXPAC), including fragile X-associated tremor/ataxia syndrome (FXTAS). The 5′ untranslated region of *FMR1* mRNA folds into a thermodynamically stable secondary structure at the region of excessively expanded CGG repeats and constitutes a template for RAN translation initiated from near-cognate start codons located upstream of the CGGs. *Cis*-regulatory elements, including sequence context and stable secondary structures within mRNA, can affect translation initiation and elongation. Here, we show that different nucleotide sequence contexts close to the near-cognate start codon affect FMRpolyG synthesis. Moreover, the distance between the near-cognate start codon and downstream stable RNA structure considerably affects the efficiency of RAN translation initiation, which is positively correlated with the number of CGG repeats. In contrast, translation elongation is impaired as CGG repeats expand. We show that native FMRpolyG containing a short polyglycine tract is synthesized efficiently but rapidly degraded by the proteasome. Our results provide insight into the structural dependencies that regulate the translation of CGGs and can be used in other repeat expansion disorders. We also show that the RNA structure is a potential therapeutic target in FXPAC.

## Graphical Abstract

**Graphical Abstract:**
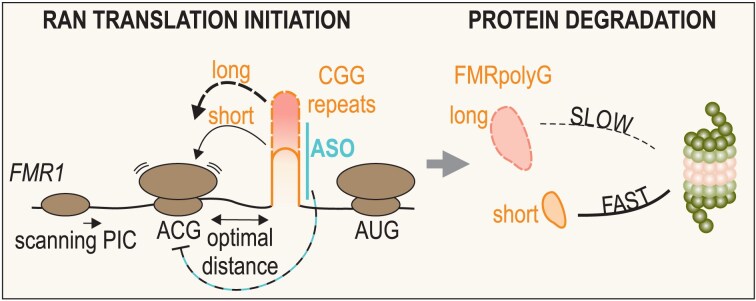

## Introduction

The expansion of short tandem repeats located in either coding or non-coding regions of different genes underlies the pathogenesis of diverse human neurological diseases known as repeat expansion disorders (REDs). REDs constitute over 50 inherited neurological disorders, including neurodevelopmental, neuromuscular, and neurodegenerative genetic conditions [1, 2].

The expansion of genetically unstable CGG repeats (known as a dynamic mutation) within the 5′ untranslated region (5′UTR) of *FMR1* has been implicated in the pathogenesis of fragile X premutation associated conditions (FXPAC). The *FMR1* gene is located on the X chromosome and encodes fragile X messenger ribonucleoprotein 1 (FMRP), a classic RNA binding protein (RBP) essential for normal brain development and synaptic plasticity [3, 4]. In the general population, the number of CGG repeats within the *FMR1* 5′UTR is highly variable; however, the majority of *FMR1* alleles have 29–30 repeats [5]. The expansion of CGG repeats (CGGexp) outside of the normal range falls into two distinct categories: the premutation (PM) with 55–200 repeats, which induce gain-of-gene function, and the full mutation (FM) with expansions greater than 200, which induce loss-of-gene function [6]. PM carriers are at risk of developing multiple FXPAC, including late-onset fragile X-associated tremor/ataxia syndrome (FXTAS) [6–8], fragile X-associated primary ovarian insufficiency (FXPOI) [9, 10], and fragile X-associated neuropsychiatric disorders (FXAND) [11]. A CGGexp to >200 is related to early onset neurodevelopmental fragile X syndrome (FXS), where the lack of FMRP reduces neural plasticity.

FXTAS is a late-onset neurodegenerative disorder caused by the limited expansion of CGG repeats (55–200). The main clinical features of the disease are intention tremor and cerebellar gait ataxia, which usually occur with several co-morbidities such as short-term memory loss, cognitive decline, parkinsonism, dementia, and autism-spectrum phenotypes [6]. FXTAS patients present an increased level of *FMR1* messenger RNA (mRNA), which correlates positively with the increasing number of CGG repeats [12–16]. Simultaneously, the increase in the number of CGG repeats correlates with a gradual impairment of the efficiency of canonical *FMR1* translation, causing a reduced level of FMRP [14, 17–19], which is not linked to FXTAS development. Interestingly, it has been shown that increased *FMR1* mRNA does not result from elevated message stability [13], as growth in the level of *FMR1* mRNA in PM carriers primarily follows from an increased transcription rate [20].

The CGGexp in the PM range within *FMR1* mRNA is directly correlated with RNA gain-of-function toxicity, where the excessively expanded CGG repeats form a thermodynamically stable secondary structure within mutant RNA that sequesters many RBPs—impairing their physiological functions involved in RNA metabolism [21–24]. Consequently, intranuclear inclusions called RNA foci are formed. Moreover, FXTAS and other FXPAC are characterized by the presence of large (2–5 µm) ubiquitin-positive intranuclear aggregates of toxic proteins produced via the non-canonical, repeat-associated non-AUG (RAN) translation [25–29] of CGG repeat tracts. The mechanism of RAN translation is based on evidence that trinucleotide repeats can be translated into protein even if they do not reside in an AUG-initiated open reading frame (ORF). Notably, RAN translation of *FMR1* has been reported to be cap-dependent [27], suggesting engagement of canonical translation initiation pathways despite initiation directly at a near-cognate start codon. In addition, translation across CGG repeats is prone to ribosomal frameshifting [30, 31], which may contribute to the diversity of RAN translation products.

The RAN translation of CGG repeats leads to the synthesis of homopolymeric cytotoxic proteins in potentially three different ORFs [32]. The most abundant—and perhaps most important for the pathology of FXPAC—is the polyglycine tract-containing protein (FMRpolyG; GGC codons in the +1 frame of FMRP), but also polyalanine- (FMRpolyA; GCG) and polyarginine tract-containing proteins (FMRpolyR; CGG) can be translated. FMRpolyG was found to be the most abundant RAN product within protein aggregates in FXTAS samples [32], while the (+2) reading frame encoding FMRpolyA is translated at only ~30% of FMRpolyG efficiency [33]. Interestingly, FMRpolyR is easily detectable only in the reporter system when no CGG repeats are present [27] and has, thus far, not been detected in pathological samples from FXTAS cases. Importantly, proteins synthesized via RAN translation have toxic properties as they are prone to aggregate and create nuclear or cytoplasmic inclusions that may sequester other proteins or affect the structure of the nuclear envelope [34]. Indeed, nearly all RAN proteins translated from expanded CGG repeats within *FMR1* 5′UTR have been reported to colocalize with ubiquitinated neuronal inclusions [28, 29, 32, 34, 35] pointing out their contribution to the neuropathology of FXTAS. However, they are not equally produced and abundant within aggregates that makes the study of RAN proteins other than FMRpolyG much more difficult. It is worth noting that many RAN proteins, synthesized from other short tandem repeats, are involved in the pathomechanisms of various REDs.

Upstream ORFs (uORFs) are mRNA sequences in the 5′UTR that are in-frame or out-of-frame with the main, downstream AUG-initiated ORF. The translation of uORFs usually results in the synthesis of short polypeptides that repress the translation of the downstream ORF [36]. The inhibition of the main downstream ORF by the uORF is usually mild because uORFs typically begin from the near-cognate start codons or the AUG start codons embedded in the poor Kozak sequence. However, this is not what is observed for the ACG (+1) near-cognate start codon [translation initiation site (TIS) for FMRpolyG], which is embedded in the optimal Kozak sequence context.

The ACG (+1) near-cognate start codon within the *FMR1* 5′UTR functions as the TIS of a uORF and is closely linked to the mechanism of RAN translation and FMRpolyG synthesis. Importantly, the ACG (+1) codon is well conserved across different species [37], suggesting that both its position and sequence context represent a biologically relevant and regulated mechanism of translational regulation. Translation initiation at this codon can be influenced by both *cis*- and *trans*-acting factors. Importantly, expanded CGG repeats downstream of ACG (+1) form stable RNA secondary structures that may act as obstacles for scanning ribosomes, increasing the dwell time of pre-initiation complex (PIC) at that codon. Such ribosome queueing may enhance initiation efficiency at the ACG (+1) near-cognate start codon, thereby promoting uORF translation and facilitating RAN translation from the *FMR1* mRNA. Notably, *trans-*acting factors, such as RNA helicases [27, 38], may also regulate FMRpolyG translation, potentially by modulating PIC scanning dynamics. Moreover, we have previously shown that the insulin-like growth factor 2 mRNA-binding protein 3 (IGF2BP3) directly binds the *FMR1* 5′UTR at a sequence motif located upstream of the CGG repeat tract, resulting in increased FMRpolyG level [39].

In light of this, and as the levels of FMRP are decreased in FXTAS, this phenomenon may result from the fact that FMRpolyG RAN translation acts as an uORF to repress downstream FMRP synthesis [37]. However, the decreased level of FMRP may also directly result from the disturbed scanning of small ribosomal subunits through expanded CGG repeats and/or diminished translation efficiency.

In this study, we evaluated the efficiency of FMRpolyG RAN translation initiation and elongation, depending on the nucleotide sequence context in the vicinity of the near-cognate ACG (+1) start codon and the role of stable secondary RNA structures formed by the sequence located downstream of the ACG (+1) codon in RAN translation initiation. In addition, we tested how different sizes of CGG repeats affect the efficiency of FMRpolyG biosynthesis. Finally, we showed a length-dependent correlation between the number of CGG repeats and the cellular stability of FMRpolyG-native proteins containing short polyglycine tracts are very unstable.

## Materials and methods

### Genetic constructs and cloning

The 16 CGG and 99 CGG constructs were previously described [40]. Briefly, these plasmids contain a 5′UTR sequence of the *FMR1* gene with either 16 or 99 CGG repeats, without any tag. The 14, 24, 38, 44, and 56 CGG constructs were obtained as a result of the repeat instability during the bacterial culture growth. The number of repeats was confirmed by Sanger sequencing. pXPG-CMV-Fluc construct was a kind gift from Michał Sekrecki and contains ORF for Firefly luciferase under the control of CMV promoter cloned on the backbone of pXPG plasmid (#71248; Addgene). 5′(CGGexp)-GFP(+1) [34] (#63091; Addgene) construct was a kind gift from Nicolas Charlet-Berguerand. Briefly, 5′(CGGexp)-GFP(+1) contains the 5′UTR of the *FMR1* gene with 99 CGG repeats and is fused to the eGFP sequence.

The Nanoluciferase (Nluc) reporter system was generated based on the pNL1.1.CMV Vector (#N1091; Promega) backbone. The FLAG tag sequence (5′-GATTACAAGGATGACGACGATAAG-3′) was added to the C-terminus of the Nluc sequence by inverse polymerase chain reaction (PCR) with F1/R1 primers. The sequence of 109 nt between the CMV promoter and the *FMR1* insert sequence was removed to ensure the transcription start site (TSS) at the beginning of *FMR1* 5′UTR. The deletion was performed by inverse PCR with the F2/R2 primers. In parallel, the *FMR1* 5′UTR containing 16 CGG repeats has been amplified from 16 CGG plasmid [40] by F3/R3 primers (+0 frame) and F4/R4 primers inserting one extra nucleotide (+G) to perform frameshift for FMRpolyG (+1 frame). Both PCR products—linearized vector and insert, were assembled and used for the transformation of NEB^®^ Stable Competent *Escherichia coli* cells (NEB). The ATG within the *FMR1* exon 1 region and ATG at the beginning of the Nluc sequence were additionally mutated to AAA and GGG, respectively, in the FMRpolyG-Nluc-FLAG construct to eliminate non-specific translation in-frame with Nluc-FLAG. The mutagenesis was performed via inverse PCR with F5/R5 primers. Each designed mutation of *FMR1* 5′UTR was cloned in parallel in two ORFs generating either FMRpolyG-Nluc-FLAG (+1 ORF) or FMRP-Nluc-FLAG (+0 ORF) constructs.

The cloning procedures were performed either according to the Gibson assembly approach, with the usage of NEBuilder^®^ HiFi DNA Assembly (NEB) and specifically designed primers having the 15 nt overhangs, complementary to the backbone or via the standard inverse PCR followed by plasmid self-circularization during ligation (Thermo Fisher Scientific). The following mutations were performed via inverse PCR with primers containing 15 nt overhangs: WK1 (F6/R6), WK2 (F7/R7), WK3, (F8/R8), SK1 (F9/R9), SK2 (F10/R10), SK3 (F11/R11), ACG > CTG (F12/R12), ACG > GTG (F13/R13), ACG > AAA (F14/R14), rACG1 (F15/R15), and rACG2 (F16/R16). For Hp mutants, the 42-nt-long sequence was selected [41] and redesigned to avoid STOP codon and CUG near-cognate start codon activity and to maintain the original hairpin stability. The hairpin-forming sequence (5′-GCCTTGGCCGGAGCGCCCGGATCCGGGCGCTCCG GCCAAGGC - 3′; ∆G = −46.1 kcal/mol) was cloned downstream of the ACG (+1) codon by following primers: Hp2nt (F17/R17), Hp6nt (F18/R18), Hp14nt (F19/R19), and Hp20nt (F20/R20). The Ins18nt mutant has inserted 18-nt-long sequence (5′-CACACACACACACACACA - 3′) 14 nucleotides downstream of the ACG (+1). The sequence was predicted to not form any secondary RNA structure (∆G = −0 kcal/mol) and was cloned via (F21/R21). All PCR products were run on the agarose gel, appropriate bands were cut, DNA was purified and assembled in a reaction with NEBuilder^®^ HiFi DNA Assembly (NEB) according to the manufacturer’s instructions. Two to five microlitres of the reaction mix were then used to transform competent cells.

The following mutations of the WT constructs were performed via standard inverse PCR with primers without overhangs: GTG > GTT mutation (F22/R22), ACG > ATG mutation (F23/R23), Del15nt (F24/R24), no CGG (F25/R25), and ACG(+0)AAA (F26/R26). Amplified PCR products were run on agarose gel, cut and purified, phosphorylated (Thermo Fisher Scientific), and used for self-circularization during ligation (Thermo Fisher Scientific). Two to five microlitres of the reaction mix were then used to transform competent cells.

To generate a construct containing a near-physiological number of CGG repeats (~30), gDNA isolated from induced pluripotent stem cells (iPSC) containing 29 CGG repeats in 5′UTR of *FMR1* was used as a template for nested PCR (F31/R31). The product of amplification was gel-purified and subsequently used as a template for a second PCR introducing an NruI restriction site (F28/R32). Obtained fragment was digested with NruI and XhoI and ligated into the previously digested FMRpolyG-Nluc-FLAG-NruI plasmid. As the pNL1.1.CMV Vector (#N1091; Promega) plasmid contains two XhoI restriction sites, digestion conditions were optimized to achieve selective cleavage at the site located downstream of the CGG repeat region.

To generate constructs with 85 CGG repeats, the 5′(CGGexp)-GFP(+1) [34] plasmid was used as the source of long CGG tract. Due to the lack of an appropriate restriction site new restriction site, NruI has been cloned directly upstream of the CGG repeats on both donor and recipient plasmids: 5′(CGGexp)-GFP(+1) and FMRPolyG/FMRP-Nluc-FLAG with 16 CGGs. The mutations were introduced by F27/R27 and F28/R28 primers, respectively. PCR products were run on the agarose gel and appropriate bands were cut out. Purified DNA was then used for ligation followed by transformation into competent cells. Next, the generated 5′(85CGG)-GFP(+1)NruI plasmid was digested with NruI and XhoI to obtain an insert containing 85 CGG repeats that was ligated to destination 16FMRpolyG/FMRP-Nluc-FLAG-NruI vectors digested with the same restriction enzymes. Digested 16FMRpolyG/FMRP-Nluc-FLAG-NruI vectors (without CGG repeats) and insert DNA containing 85 CGG repeats were then assembled in ligation followed by competent cells’ transformation. To avoid PCR amplification through CGG repeats, the 5′UTR regions of *FMR1* containing desired mutations (constructs already prepared with 16 CGG repeats) were used as templates for PCR. The forward primer (F29) contained the NdeI restriction site that was natively present within the CMV promoter of all constructs. The reverse primer (R29) introduced the NruI restriction site in amplicons. After amplification PCR products were purified and digested with NdeI and NruI enzymes. Simultaneously, FMRpolyG/FMRP-Nluc-FLAG-NruI plasmids with 85 CGGs were digested with the same enzymes. Both DNA fragments were purified and assembled in ligation. The sequences of all used primers are listed in Supplementary Table S1.

The FMRpolyG-Nluc-FLAG constructs with 50 and 76 CGG repeats were obtained as a result of the repeat instability during the bacterial culture growth. The CGG repeat number was verified by Sanger sequencing.

### Polymerase chain reaction

All mutations were performed via inverse PCR using either CloneAmp HiFi PCR Premix (Takara Bio) or Phusion High Fidelity Polymerase (Thermo Fisher Scientific). The optimized protocol using Phusion High Fidelity Polymerase was also utilized to amplify the DNA template for *in vitro* transcription. The reaction mix for CloneAmp polymerase contained: 1× CloneAmp HiFi PCR Premix, 0.2 µM forward and reverse primers, 5 ng of template DNA, and 1.85 M Betaine. The reaction mix for Phusion polymerase contained: 1× Phusion GC Buffer, 0.2 mM dNTP, 0.5 µM forward and reverse primers, 5 ng of template DNA, 0.4 U of polymerase, 3% dimethyl sulfoxide (DMSO), and 1.5 M Betaine.

### Cell culture and transfection

The HEK-293 cells were grown in a high glucose Dulbecco’s modified Eagle’s medium (DMEM) supplemented with L-glutamine (Biowest), 10% fetal bovine serum (Biowest), and 1% antibiotic/antimycotic (Sigma). S-16CGG and S-99CGG cells were grown in a DMEM containing certified tetracycline-free FBS (Biowest) and were treated with doxycycline to induce transgene expression as described in [38]. All cells were grown at 37°C in a humidified incubator containing 5% CO_2_. Transfections were performed with the use of Lipofectamine 3000 (Thermo Fisher Scientific) according to the manufacturer’s instructions. For transient expression analysis, HEK-293 cells were transfected with FMRP/FMRpolyG-Nluc-FLAG plasmids and harvested 24 h post-transfection. For antisense oligonucleotide (ASO) treatment 200 nM of ASOs were delivered to HEK-293 cells 3 h post-transfection with appropriate construct and cells were collected 48 h post ASO treatment. For the Nluc assay, HEK-293 cells were co-transfected with 20 ng of FMRP/FMRpolyG-Nluc-FLAG plasmids and 100 ng of pXPG-CMV-Fluc construct. For proteasome inhibition, HEK-293 cells were transfected with plasmids containing 14, 24, 38, 44, 56, and 99 CGG repeats (without any tag). Twelve hours post-transfection, cells were treated with 5 µM MG132 or DMSO for 6 h. In a separate experiment, HEK-293 cells transfected with 24 or 99 CGG repeat plasmids (untagged) were treated 12 h post-transfection with 200 nM Botezomib or DMSO for 6 h.

### iPSC culture

The procedure of iPSC culture has been previously described in [39]. Briefly, one human FXTAS iPSC line, expressing 72 CGG repeats in the *FMR1* 5′UTR, was a kind gift from Nicolas Charlet-Berguerand, and was described earlier [34]. Legal ethical approval and informed consent from the patients were obtained to generate and reuse these iPS cells for scientific and research purpose. One human control iPSC line, harboring 29 CGG repeats in the 5′UTR of *FMR1* was reprogrammed in our laboratory using Episomal iPSC Reprogramming Vectors (Thermo Fisher Scientific). iPSCs were cultured in StemFlex (Thermo Fisher Scientific) medium on Geltrex-coated (Thermo Fisher Scientific) plates at 37°C humidified incubator containing 5% CO_2_. Cells were passaged every 3–4 days using PBS–ethylenediaminetetraacetic acid. Cell lines were routinely tested for mycoplasma using mycostrip test (Invivogen) and found negative.

### iPSC neuronal differentiation and apoptosis assay of FXTAS iPSC-derived neurons

iPSCs were differentiated into neurons as described previously [34] with modifications. Briefly, 2 million iPSCs were plated on one Geltrex-coated well (Thermo Fisher Scientific) of 6-well plate in Essential 8 medium (Gibco) with 10 µM Y-27632 dihydrochloride (MedChemExpress). The next day, medium was changed to NFS medium [N2B27 supplemented with 20 ng/mL FGF2 (Peprotech), 0.25 µM LDN-193189 (Sigma), and 10 µM SB431542 (MedChemExpress)], and cells were grown for 10 days with daily medium change. After the appearance of neural rosette, cells were passaged onto poly-L-ornithine (Merck) and laminin (Merck)-coated 96-well plates, and NFS medium was replaced with NSC medium [N2B27 supplemented with 20 ng/mL hBDNF (Peprotech), 1 µM LY-411575 (MedChemExpress), and 0.2 µM Ascorbic Acid (Sigma)]. Media change was performed every two days and cells were differentiated for 10 days in NSC medium.

At day 11, ASO-3′, ASO-SCR, ASO-CCG, and ASO-Ctrl were delivered to cells by lipofection using RNAiMAX (Thermo Fisher Scientific). Forty-eight hours post-ASO delivery, necrosis was tested with RealTime-Glo™ Annexin V Apoptosis and Necrosis Assay (Promega) according to the manufacturer’s instructions. Fluorescence signal corresponding to necrosis was measured at different time points starting from 1 h after addition of the reaction mix, using SPARK microplate reader (TECAN).

### Antisense oligonucleotides

ASOs were synthesized and HPLC purified by Kaneka Eurogentec. ASO-3′ (5′-GCGCTCGAGGCCCAGCCGCC - 3′) and ASO-SCR (5′-GCCGGACGCCACGCTCGCGC - 3′) were 20-nucleotide-long and were exclusively composed of 2′-methoxyethyl units. All positions were phosphorothioated. ASO-CCG (5′-CCGCCGCCG - 3′) and ASO-Ctrl (5′-TGAACATAA-3′) were 9-nucleotide-long oligonucleotides composed of 8 locked nucleic acid (LNA) units and a 2′-O-Me unit at 3′ end. All LNA positions were phosphorothioated. In all experiments, ASOs were denatured before transfection for 30 s at 95°C and chilled on ice. The ASO-3′ is the subject of a patent application (no. P.455445).

### Nano-Glo dual-luciferase reporter assay

For Nano-Glo Dual-Luciferase Reporter Assay (Nluc assay) HEK-293 cells were lysed 24 h post-transfection in 100 µl of RIPA buffer (Sigma) and incubated for 30 min on ice. Twenty microlitres of lysate from each sample were transferred on a black 96-well plate (Thermo Fisher Scientific), mixed with 60 µl of phosphate-buffered saline (PBS), equilibrated to room temperature, and proceeded with Nano-Glo Dual-Luciferase Reporter Assay (Promega). Nluc was normalized to Fluc to control for variation in transfection efficiencies.

### RNA isolation and quantification

The isolation of total RNA from cells was performed using TRI Reagent (Thermo Fisher Scientific) and Total RNA Zol-Out™ D kit (A&A Biotechnology). During purification on columns, the RNA was digested by the DNase. Finally, 500–1000 ng of the total RNA was used for reverse transcription (RT) with GoScript Reverse Transcriptase (Promega) and random primers (Promega) according to the manufacturer’s protocol. RT-qPCR reactions were performed using Maxima SYBR Green/ROX qPCR Master Mix (Thermo Fisher Scientific) according to the manufacturer’s instructions in QuantStudio 7 Flex Real-Time PCR System (Thermo Fisher Scientific). Ct values were normalized against GAPDH. Fold differences in expression level were calculated according to the 2−ΔΔCt method [42]. The sequences of used primers are listed in Supplementary Table S1.

### Western blot

Cells were lysed for 30 min on ice in RIPA buffer (Sigma) supplemented with 1x Halt Protease Inhibitor Cocktail (Thermo Fisher Scientific) followed by sonication and centrifugation at 10 000 *g* for 10 min at 4°C. An equal volumes of protein extracts were denatured in Bolt LDS Sample buffer (Thermo Fisher Scientific) mixed with Bolt Reducing agent (Thermo Fisher Scientific) at 95°C for 5 min. Electrophoresis was performed in a Mini Gel Tank (Invitrogen) using Bolt 4%–12% Bis–Tris Plus gel (Thermo Fisher Scientific) in Bolt MES SDS Running Buffer (Thermo Fisher Scientific). Proteins were electroblotted to PVDF transfer membrane (0.2 μm, GE Healthcare) for 1 h, at 100 V in an ice-cold Bolt Transfer Buffer (Thermo Fisher Scientific) supplemented with 10% methanol.

Membranes were blocked in 5% non-fat dry milk (Sigma) in Tris-buffered saline (TBS) with 0.1% Tween 20 (TBS-T) overnight at 4°C or at least for 1 h at RT. Incubation with the following antibodies: rabbit anti-FMRP (ab17722, Abcam) 1:1000, mouse anti-FMRpolyG (9FM; recognizes C-terminal part of FMRpolyG [29]; MABN1788, Sigma) 1:1000, and mouse anti-β-catenin (sc-7963, Santa Cruz Biotechnology) 1:1000 was performed in 5% non-fat dry milk in TBS-T overnight at 4°C. Mouse anti-GAPDH HRP-conjugated antibody (sc-47724, Santa Cruz Biotechnology) 1:10 000, mouse anti-vinculin HRP-conjugated antibody (sc-73614 HRP, SantaCruz Biotechnology) 1:5000, and mouse anti-FLAG HRP-conjugated antibody (A8592, Sigma) 1:10 000 were diluted in 5% non-fat dry milk in TBS-T, and incubated for 1 h at RT. Membranes were washed in TBS-T and incubated with horseradish peroxidase-conjugated secondary antibodies (not applicable for HRP-conjugated primary antibodies): anti-rabbit (A9169, Sigma) 1:10 000 or anti-mouse (A9044, Sigma) 1:10 000 for 1 h at RT and washed with TBS. Antibody–antigen complexes were visualized by enhanced chemiluminescence using Immobilon Forte Western HRP substrate (Sigma) and detected with G:BoxSystem (Syngene) or ChemiDoc Imaging System (BioRad). Intensities of the protein signals were measured and quantitated with GeneTools 4.02 (Syngene) or ImageLab (BioRad), respectively. For detection of the signal from different antibodies, the membrane was cropped or washed with stripping buffer [1.5% glycine, 0.1% sodium dodecyl sulfate (SDS), 1% Tween 20, pH 2.2].

### RNA secondary structure predictions

Predictions of secondary structures formed by single-stranded RNA sequences were performed using the RNAfold WebServer (http://rna.tbi.univie.ac.at/cgi-bin/RNAWebSuite/RNAfold.cgi). Folding simulations were conducted at 37°C to approximate physiological conditions and structures were predicted based on the minimum free energy and base pair probabilities.

### *In vitro* transcription

Templates for *in vitro* transcription were generated by PCR (F30/R30; Phusion™ High-Fidelity DNA Polymerase; Thermo Fisher Scientific) and purified using Clean-Up Concentrator (A&A Biotechnology). The PCR products were transcribed into capped RNA using an mMESSAGE mMACHINE T7 Transcription Kit (Thermo Fisher Scientific). The reaction was assembled according to the manufacturer’s instructions and performed at 37°C for 1.5 h. The transcribed RNA was treated with Turbo DNase (Thermo Fisher Scientific) and purified using a Clean-Up RNA Concentrator (A&A Biotechnology).

### *In vitro* translation

*In vitro* translation was performed as described by Susorov *et al*. 2020 [43] with some modifications. The reaction mixture was assembled on ice and contained 50% of nuclease-treated rabbit reticulocyte lysate (Promega), 30 mM Hepes-KOH (pH 7.5), 50 mM KCl, 1 mM Mg(OAc)_2_, 0.2 mM ATP, 0.2 mM GTP, 0.02 mM amino acid mix minus leucine, 0.02 mM amino acid mix minus methionine (Promega), 2 mM dithiothreitol (DTT), 1% Nluc substrate furimazine (Promega), and 10 nM RNA. When necessary, ASO was added to the mixture at 100 nM concentration. RNA was denatured (65°C for 3 min) immediately prior to *in vitro* translation. Fifteen microlitres aliquots of the mixture were placed in a 384-well plate. Reactions were performed at 30°C. The luminescence signal was recorded every 45 s over a period of 30 min using a TECAN Spark plate reader. For analysis, the growing linear section of the luminescence curve indicating the maximum values of the luminescence signal over time (ΔRLU/min) was used.

### Statistics and reproducibility

All data were processed and analysed using Microsoft Excel. Statistical analysis was performed using multiple unpaired parametric *t*-tests with Holm–Šídák correction for multiple comparisons or an unpaired two-sided *t*-test. Tests that resulted in *P* < .05 have been reported to be statistically significant. The symbols; *, **, ***, **** represent values of *P* < .05, *P* < .01, *P* < .001, and *P* < .0001, respectively. All data were analysed using GraphPad Prism software. Error bars represent standard deviation (SD). All cellular and *in vitro* experiments were repeated independently at least two times with similar results and indicated ‘N’ represents individual biological replicates in the same experiment.

## Results

**Figure 1.**
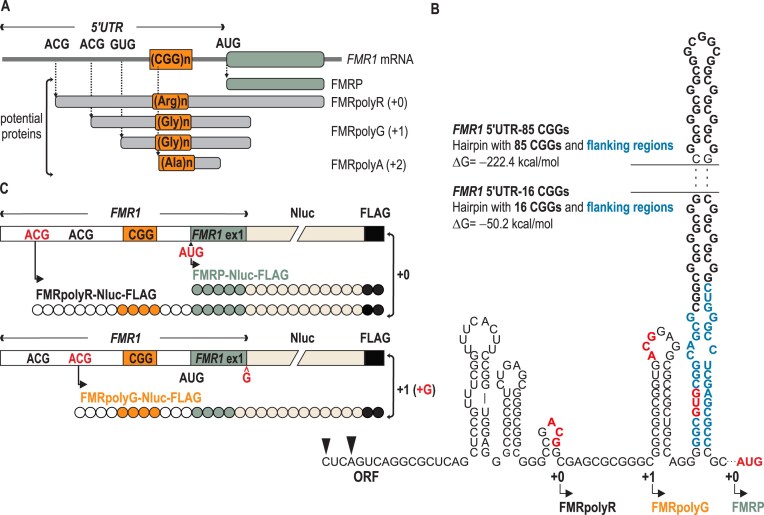
Scheme of *FMR1* mRNA and RAN translation products. (**A**) *FMR1* mRNA is a template for the synthesis of at least four different proteins: the AUG codon downstream 5′UTR is used to produce FMRP. Three near-cognate start codons in the 5′UTR allow for the initiation of biosynthesis of the following RAN products: FMRpolyR [ACG (+0)] and FMRpolyG [ACG (+1) and GUG (+1)]. Initiation within the CGG repeats generates FMRpolyA [GCC (+2)]. (**B**) Predicted secondary structure of *FMR1* 5′UTR with 16 and 85 CGG repeats. The predicted RNA structure stability of CGG-derived hairpins with flanking sequences expressed as ∆G values are presented. The near-cognate and canonical start codons are marked in red. The flanking regions experimentally shown to stabilize the CGG hairpin [44] are highlighted in blue. Two black arrowheads correspond to the estimated TSSs. (**C**) Scheme of generated constructs and their protein products. Two ACG near-cognate start codons are shown (+0 and +1, respectively), and CGG repeats are marked in orange. The *FMR1* ex1 sequence (AUG +0) encoding for FMRP is marked in green. In this model FMRP equivalent is composed of 17 aa of FMRP fused to Nluc-FLAG (FMRP-Nluc-FLAG protein). The insertion of the G residue to generate frame shift to the FMRpolyG fused with Nluc (FMRpolyG-Nluc-FLAG protein) is shown in red.

Three specific near-cognate start codons have been established as donors for RAN proteins from the 5′UTR of *FMR1* mRNA (Fig. 1A). The +0 frame initiates at the ACG near-cognate codon 57 nt upstream of the CGG repeats and produces FMRpolyR (N-terminal polyarginine extension of FMRP). The +1 FMRpolyG can be initiated from ACG or GUG near-cognate start codons located 32- and 8-nt upstream of the CGGs, respectively. To determine *cis*-acting factors regulating the RAN translation initiation of FMRpolyG, we generated a series of genetic constructs expressing a fragment of human *FMR1* mRNA containing the entire 5′UTR with either 16 or 85 CGGs and the sequence encoding for the N-terminal part of FMRP fused to a sequence encoding Nluc tagged with FLAG at the C-terminus. The distance between the promoter and the *FMR1* sequence was designed to ensure that transcription started at the beginning of the native 5′UTR sequence (Fig. 1B; black arrowheads). Each *FMR1* sequence variant was cloned in parallel in two reading frames of the luciferase reporter (Fig. 1C). First, the FMRP-Nluc-FLAG allowed us to monitor the level of the +0 FMRP equivalent −FMRP-Nluc-FLAG protein (first 17 aa of FMRP fused with the Nluc-FLAG) as a control of AUG-initiated canonical translation and +0 FMRpolyR −FMRpolyR-Nluc-FLAG protein (Fig. 1C; upper panel). Second, the FMRpolyG-Nluc-FLAG allowed us to measure the production of FMRpolyG initiated from the +1 reading frame −FMRpolyG-Nluc-FLAG protein (Fig. 1C; lower panel). The predictions of *FMR1* 5′UTRs’ secondary structures revealed that the number of CGG repeats may not affect RNA secondary structures in the vicinity of the repeat tract. However, it has a strong influence on the thermodynamic stability of the hairpin structure formed by the CGG repeats and adjacent nucleotides (for 16CGG hairpin the ΔG = −50.2 kcal/mol, and for 85CGG hairpin the ΔG = −222.4 kcal/mol) (Fig. 1B). These structures can significantly affect both translation initiation and elongation.

RAN translation of FMRpolyG is initiated mainly from ACG (+1) [26, 34]. Therefore, to ensure translation from a single, particular codon, we performed a mutagenesis of the second near-cognate start codon—GUG (+1) (Supplementary Fig. S1Aa). A GTG>GTT mutation (GUG to GUU codon) confirmed the results published in another study showing that the GUG (+1) codon plays a minor role in the initiation of FMRpolyG synthesis under non-stressed conditions [27, 45], as no changes in the level of FMRpolyG-Nluc-FLAG and the FMRP-Nluc-FLAG were observed (Supplementary Fig. S1Ab and Ac). The introduced mutation did not affect the predicted structure of the *FMR1* 5′UTR (Supplementary Fig. S1Ba and Bb), and the mRNA level remained unchanged (Supplementary Fig. S1Ad). For simplicity and greater readability, the control constructs with the native ACG (+1) near-cognate start codon and GTG>GTT mutation are named WT in all further analyses.

FMRpolyR is initiated at the ACG (+0) near-cognate start codon located upstream of the CGG repeats and the ACG (+1) codon for FMRpolyG and is an N-terminal polyarginine fused to FMRP (Fig. 1A). Since the TIS of FMRpolyR is located upstream of the major initiation site for FMRpolyG, there is a possibility that the overlapping uORF of FMRpolyR impairs translation of downstream FMRpolyG. To verify this, we performed a mutation of the ACG (+0) codon to AAA [ACG(+0)AAA mutation] (Supplementary Fig. S1Ca, Da, and Db). A western blot (WB) analysis confirmed that the observed extra protein migrating above the FMRP-Nluc-FLAG was FMRpolyR-Nluc-FLAG, as the introduced mutation resulted in protein loss (Supplementary Fig. S1Cb; left panel). As expected, the level of the FMRP-Nluc-FLAG remained unchanged for both 16 and 85 CGGs containing mRNAs (Supplementary Fig. S1Cb; middle panel, Cc, and Cd). Similarly, no significant changes in the protein level in the (+1) frame were observed. The unchanged level of FMRpolyG-Nluc-FLAG upon the ACG(+0)AAA mutation indicates that RAN translation in the polyR-frame does not contribute to translation in the polyG-frame in our model system.

### Different near-cognate start codons within the 5′UTR of *FMR1* can effectively initiate RAN translation of FMRpolyG

**Figure 2.**
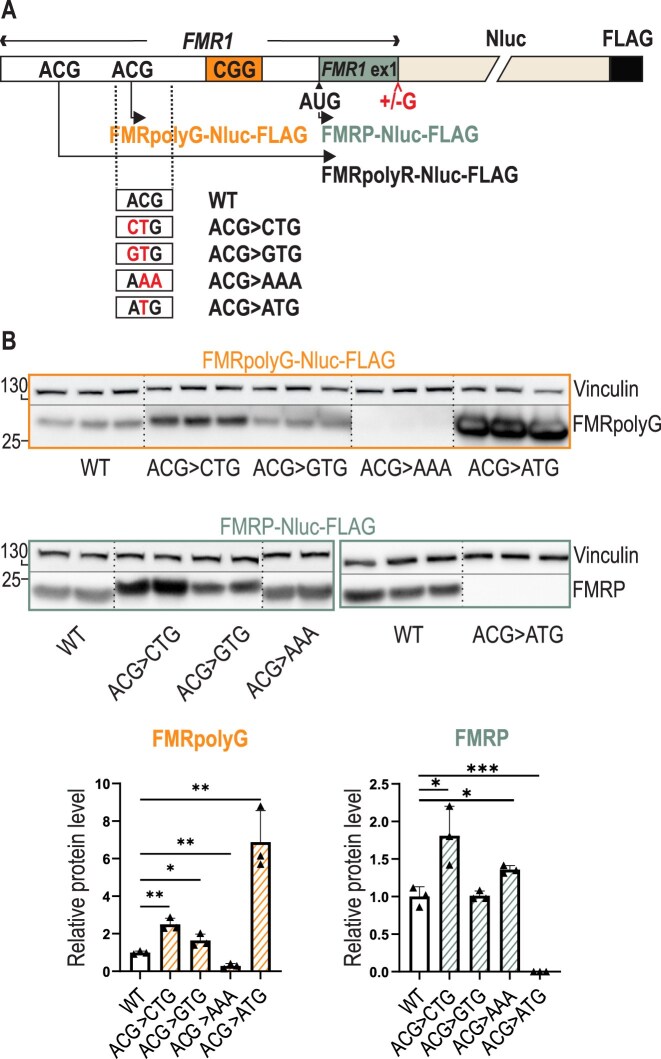
The efficiency of FMRpolyG translation initiation at different near-cognate start codons. (**A**) Scheme of cloned constructs. Four mutants of ACG (+1) near-cognate start codon have been designed: ACG>CTG (CUG codon), ACG>GTG (GUG), ACG>AAA, and ACG>ATG (AUG). (**B**) WB analysis and corresponding quantification of FMRpolyG-Nluc-FLAG and FMRP-Nluc-FLAG levels in HEK-293 cells 24 h post-transfection with indicated constructs. Results were normalized to Vinculin. Graphs represent averages for *N* = 3 biologically independent samples with SDs relative to WT = 1. Gels were cropped (dashed lines); statistical analysis was performed using multiple unpaired parametric *t*-tests with Holm–Šídák correction for multiple comparisons; *P < .05; **P < .01; ***P < .05.

While most near-cognate start codons have been shown to initiate translation at very low frequencies, some codons can support high levels of translation initiation with efficiencies up to 50% of the AUG codon in the optimal Kozak sequence context [46]. To test the efficiency of RAN translation initiation of FMRpolyG from different near-cognate start codons embedded in the same sequence context, we designed four mutants containing the following mutations of the ACG (+1) codon: ACG>CTG, ACG>GTG, ACG>AAA (which does not support initiation), and ACG>ATG (which supports canonical translation initiation) (Fig. 2A and Supplementary Fig. S2a).

Both ACG>CTG and ACG>GTG mutations increased the efficiency of FMRpolyG-Nluc-FLAG translation initiation visualized by WB (Fig. 2B and Supplementary Fig. S2b). However, the CUG codon was more efficiently utilized. We assumed that the ACG>AAA mutation would not support initiation and, hence, scanning PICs would bypass the FMRpolyG frame and initiate only at the downstream AUG start codon of the FMRP-Nluc-FLAG. Indeed, the mutation resulted in the complete loss of the FMRpolyG-Nluc-FLAG protein (Fig. 2B and Supplementary Fig. S2b). As the ACG>ATG mutation led to a high increase in translation initiation (measured by the level of FMRpolyG-Nluc-FLAG protein), the extensive growth in the efficiency of translation initiation from the mutated AUG codon had a direct effect on the signal loss of the FMRP-Nluc-FLAG. In contrast, the ACG>AAA mutation and, surprisingly, the ACG>CTG mutation slightly but significantly increased the level of the FMRP-Nluc-FLAG. Of note, the reporter mRNAs from all constructs remained at a comparable level (Supplementary Fig. S2c).

Together, our experiments confirmed that other near-cognate start codons, such as CUG and GUG in the same Kozak sequence context, are effective in the RAN translation initiation of FMRpolyG-Nluc-FLAG. Moreover, since both FMRpolyG and FMRP proteins are translated natively from the same mRNA, the results of the ACG>ATG mutation suggest that all scanning PICs were captured by the mutated AUG codon and did not allow for FMRP-Nluc-FLAG synthesis.

### Kozak sequence context influences the initiation of FMRpolyG RAN translation

The particular nucleotides surrounding a start codon strongly affect the efficiency of initiation across various vertebrate mRNAs [47]. These nucleotides are commonly referred to as the ‘Kozak sequence’. The optimal Kozak sequence in vertebrates is GCCRCCATGG, where R is a purine (A or G) [48]. Positions −3 and +4 (where +1 refers to A in AUG) are considered the most important for regulating the efficiency of the translation initiation process due to their stabilizing interactions with the PIC [49]. Importantly, these surrounding nucleotides have a larger influence on the recognition of non-AUG start codons than on canonical AUG start codons [46].

**Figure 3.**
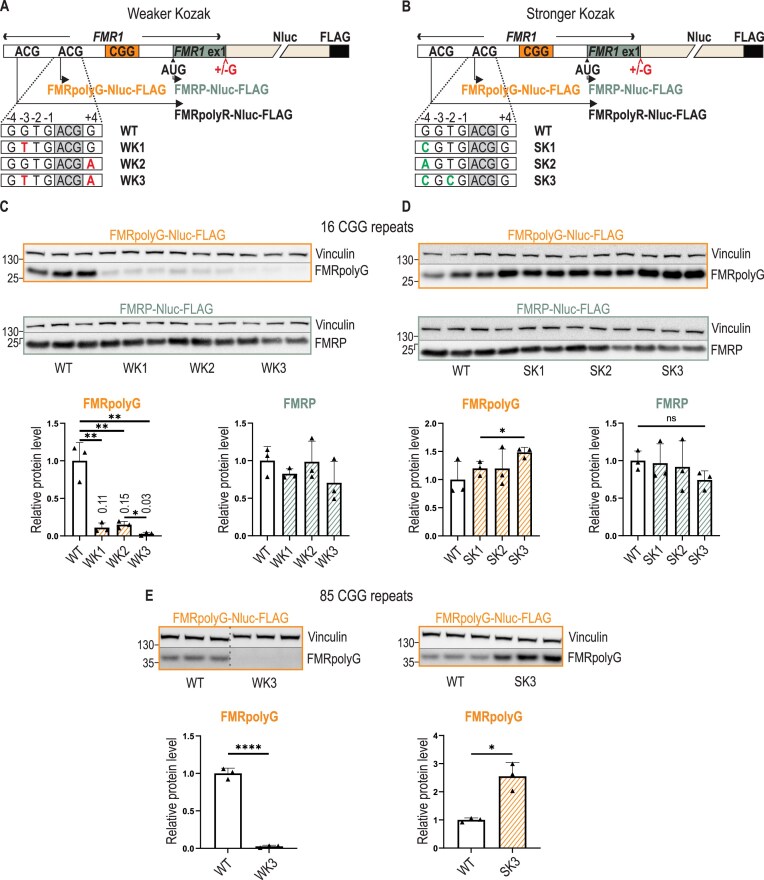
The efficiency of FMRpolyG translation initiation at ACG (+1) near-cognate start codon embedded within a modified context of Kozak sequence. (**A, B**) Schematics of cloned constructs. Three mutants of ACG (+1) Kozak sequence were designed to make the context weaker: WK1, WK2, and WK3, and three mutants were designed to make the context stronger: SK1, SK2, and SK3. WB analyses and corresponding quantifications of FMRpolyG-Nluc-FLAG and FMRP-Nluc-FLAG levels in HEK-293 cells 24 h post-transfection with (**C**) weaker WK1-3 and (**D**) stronger SK1-3 context for constructs with 16 CGGs. (**E**) WB analyses and corresponding quantifications of FMRpolyG-Nluc-FLAG levels in HEK-293 cells 24 h post-transfection with WT and WK3 (left) or WT and SK3 (right) constructs containing 85 CGGs. In panels (C–E), the results were normalized to Vinculin. Graphs represent averages for *N* = 3 biologically independent samples with SDs relative to WT = 1; statistical analysis was performed using multiple unpaired parametric *t*-tests with Holm–Šídák correction for multiple comparisons; *P < .05; **P < .01; ****P < .0001; ns, non-significant.

To study how the Kozak sequence affects the efficiency of FMRpolyG translation initiation, we designed 6-nucleotide context mutants around the ACG (+1) near-cognate start codon of mRNAs harbouring short, 16 CGG repeats. Three of them were designed to weaken the Kozak sequence (WK1, WK2, and WK3; Fig. 3A and Supplementary Fig. S3Aa), and another three were designed to make the context stronger (SK1, SK2, and SK3; Fig. 3B and Supplementary Fig. S3Ab). WK mutations at the −3 and +4 positions (WK1 and WK2, respectively) significantly reduced RAN translation initiation, and for a double WK3 mutant, FMRpolyG-Nluc-FLAG was barely detected (Fig. 3C and Supplementary Fig. S3Ba). SK mutations at the −4 position (SK1 and SK2) or both −4 and −2 positions (SK3) slightly increased RAN translation, as native ACG (+1) was almost in an optimal Kozak context (Fig. 3D and Supplementary Fig. S3Bb). As expected, these mutations did not influence the initiation of canonical translation as measured by the synthesis of the FMRP-Nluc-FLAG (Fig. 3C and D, and Supplementary Fig. S3Ba and Bb).

One of the explanations of the RAN translation mechanism assumes that translation initiation at near-cognate start codons is stimulated by paused scanning PICs and/or translating ribosomes, which meet an RNA secondary structure obstacle formed by expanded CGG repeats. Thus, a formed queue of stacked ribosomes/PICs and increased dwell time of PIC at the near-cognate start codon may favour the initiation of uORF translation even if their start codons are embedded in the weak Kozak sequence context. Therefore, we wanted to check whether a thermodynamically stable RNA secondary structure formed by 85 CGG repeats would force or increase the initiation at the ACG (+1) near-cognate start codon lying in the weakest (WK3) or the strongest (SK3) Kozak sequence context, respectively. The stable structural obstacle formed by 85 CGG repeats did not increase the level of FMRpolyG-Nluc-FLAG in the WK3 mutant (Fig. 3E, left). However, it significantly raised the level of RAN protein in the SK3 mutant (Fig. 3E, right).

These results suggest that sequence composition has a greater effect on the efficiency of RAN translation initiation when the Kozak sequence context is disturbed. However, the efficiency of FMRpolyG-Nluc-FLAG translation initiation from ACG (+1) in an optimal Kozak sequence can be slightly strengthened by downstream (and extremely stable) secondary RNA hairpin structure formed by 85 CGG repeats.

### Distance between native ACG (+1) and downstream stable RNA secondary structure significantly affects RAN translation initiation

**Figure 4.**
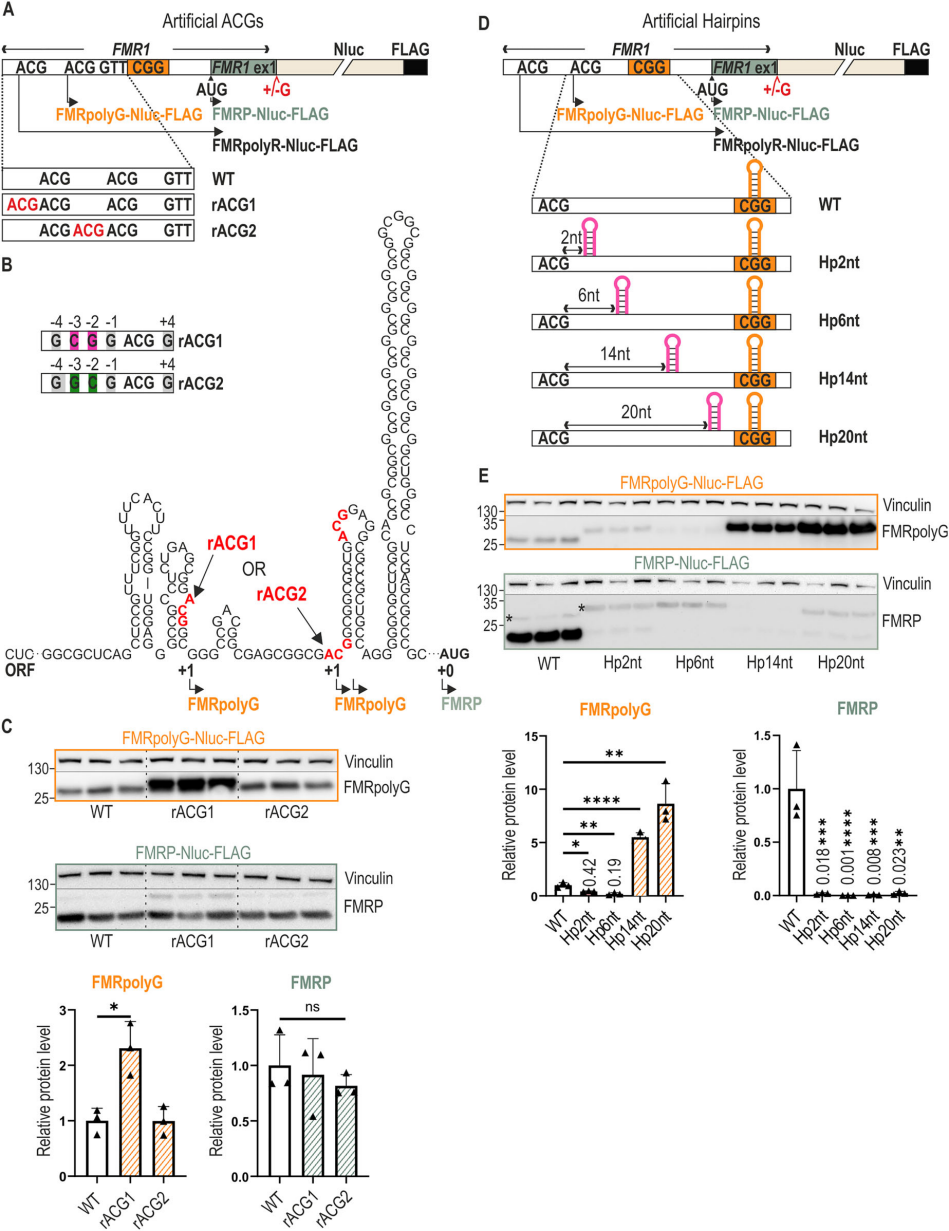
The RNA structure-dependent efficiency of FMRpolyG translation initiation. (**A**) Scheme of cloned artificial random rACG1- and rACG2-containing constructs. Additional ACG (+1) near-cognate start codons (marked in red) were cloned either upstream of the native ACG (+0) codon (rACG1) or upstream of the native ACG (+1) codon (rACG2). (**B**) Predicted secondary RNA structure of *FMR1* 5′UTR with introduced mutations. Both mutations are presented simultaneously. The Kozak sequence contexts for additional ACG (+1) near-cognate start codons are presented: the weaker Kozak context of the rACG1 (upper case), and the stronger context of the rACG2 (lower case). (**C**) WB analysis and corresponding quantification of FMRpolyG-Nluc-FLAG and FMRP-Nluc-FLAG levels in HEK-293 cells 24 h post-transfection with indicated constructs. Gels were cropped (dashed lines). (**D**) An artificial hairpin-forming sequence was cloned at different regions downstream of the native ACG (+1) near-cognate start codon. (**E**) WB analysis and corresponding quantification of FMRpolyG-Nluc-FLAG and FMRP-Nluc-FLAG levels 24 h post-transfection with indicated constructs. The asterisk indicates the FMRpolyR-Nluc-FLAG protein translated from the FMRP-Nluc-FLAG construct. In panels (C–E) results were normalized to Vinculin. Graphs represent averages for *N* = 3 biologically independent samples with SDs relative to WT = 1; statistical analysis was performed using multiple unpaired parametric *t*-tests with Holm–Šídák correction for multiple comparisons; *P < .05; **P < .01; ***P < .001; ****P < .0001.

Since many near-cognate start codons within the *FMR1* 5′UTR may act as the TIS of FMRpolyG, we wanted to establish whether randomly localized ACG codons in the +1 frame would be efficient in RAN translation initiation. Two mutant RNAs with random ACG (rACG) codons were designed: rACG1 located 33 nt upstream of the native ACG (+1) codon, and rACG2 with a near-cognate start codon located 9 nt upstream of the native ACG (+1) (Fig. 4A). In both cases, the native ACG (+1) codon remained unchanged, and the predicted general structures of *FMR1* 5′UTRs remained unaffected (Fig. 4B). As expected, two proteins were produced from the rACG1 mutant: one translated from the new rACG1 (+1) codon and the other from the native ACG (+1) (Fig. 4C). The quantification of signal intensities revealed that the new ACG (+1) codon within the rACG1 mutant was effectively used given that the total level of FMRpolyG-Nluc-FLAG was twice as high as in the control (Fig. 4C and Supplementary Fig. S4Aa). Interestingly, the additional ACG (+1) codon in the second rACG2 appeared inactive, as only FMRpolyG-Nluc-FLAG translated from the native ACG (+1) was observed. Surprisingly, the observed results were inconsistent with the strength of the Kozak sequence context of both the rACG1 and rACG2 mutants, as rACG1 was characterized by a weaker Kozak sequence context than rACG2, which appeared to be inactive (Fig. 4B).

According to the scanning model of translation initiation, RNA structures located either upstream or downstream of the start codons can influence the efficiency of initiation by influencing PIC movement. Therefore, the initiation from the near-cognate start codon or the AUG start codon located in the poor Kozak sequence context can be increased when a stable secondary structure is placed downstream of the start codon [50, 51]. Due to observed discrepancies in the rACG mutants (no correlation between Kozak context and translation initiation), we studied whether different localization of ACGs within the context of RNA structures could affect RAN translation initiation.

The distance between the start codon and the downstream secondary structure is important as it directly results in the positioning of stalled PIC on the mRNA. It has been shown, based on the known size of ribosomes, that a distance of ~14 nt allows for start codon positioning in the P-site of the 43S ribosome [50, 52]. With this in mind, we tested how the distance between the native ACG (+1) and the stable secondary RNA structure influences the efficiency of FMRpolyG biosynthesis. We designed four mutants on the backbone of the mRNA with 16 CGG repeats that had inserted an artificial RNA hairpin-forming sequence at different positions downstream of the native ACG (+1). The hairpin-forming sequence had similar predicted thermodynamic stability (∆G = −46 kcal/mol) to the hairpin formed by 16 CGG repeats with stabilizing flanking regions (∆G = −50.2 kcal/mol) and was introduced 2-, 6-, 14-, and 20-nt downstream of the native ACG (+1) near-cognate start codon (Fig. 4D and Supplementary Fig. S4B).

Our experiments showed that a distance of 2 and 6 nt between the TIS and the artificial hairpin structure had a strongly negative effect on FMRpolyG-Nluc-FLAG translation initiation at ACG (+1) as these distances are too short to position the PIC on the ACG (+1) (Fig. 4E and Supplementary Fig. S4Ab). However, extending this distance to 14 and 20 nt resulted in an extremely high increase in FMRpolyG-Nluc-FLAG production, with a maximum value for the Hp20nt mutant (a ca. 10-fold increase was observed). Of note, the efficiency of FMRP-Nluc-FLAG translation was decreased in all tested mutants, which did not result from the altered level of *FMR1* mRNA (Supplementary Fig. S4Ac).

These data indicate that the distance between the particular near-cognate start codon and the obstacle formed by a stable secondary RNA structure that could slow down the scanning PIC is a very important factor influencing the efficiency of translation initiation at the ACG (+1) codon of *FMR1* mRNA.

### The location of the hairpin structure formed by CGG repeats regarding the native ACG (+1) near-cognate start codon is crucial for RAN translation initiation

**Figure 5.**
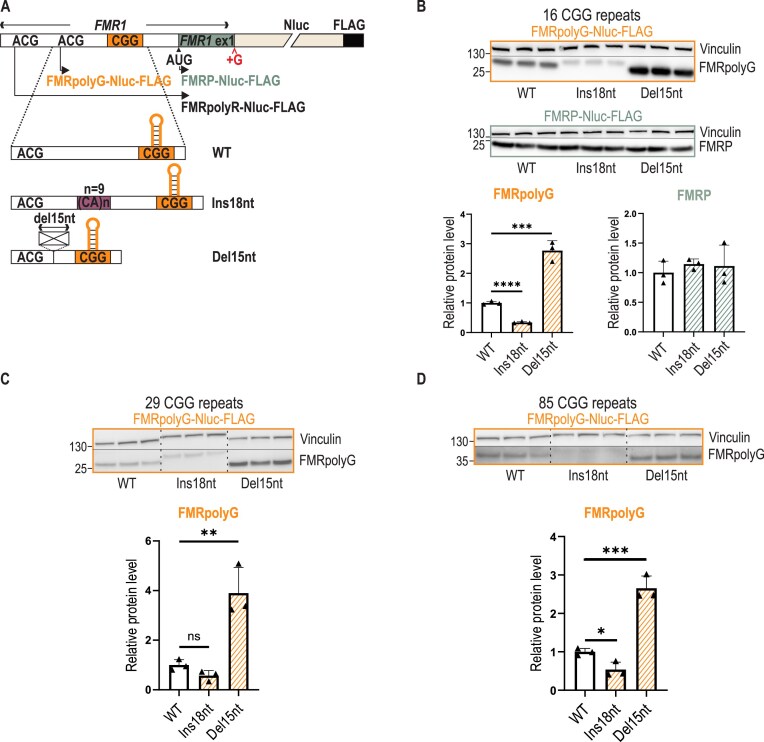
Distance between the ACG (+1) near-cognate start codon and the CGG-hairpin structure has a considerable effect on the FMRpolyG translation initiation. (**A**) Scheme of cloned constructs. The mutants with extended (Ins18nt) and shortened (Del15nt) distance between ACG (+1) and CGG repeats were designed. (**B**) WB analysis and corresponding quantification of FMRpolyG-Nluc-FLAG and FMRP-Nluc-FLAG levels in HEK-293 cells 24 h post-transfection with indicated constructs containing 16 CGGs. WB analysis and corresponding quantification of FMRpolyG-Nluc-FLAG level in HEK-293 cells 24 h post-transfection with indicated constructs containing 29 CGGs (**C**) and 85 CGGs (**D**). Gels were cropped (dashed lines); statistical analysis was performed using multiple unpaired parametric *t*-tests with Holm–Šídák correction for multiple comparisons; *P < .05; **P < .01; ***P < .001; ****P < .0001; ns, non-significant.

To move towards a more natural scenario without using artificial RNA hairpin structures, we performed two sets of experiments using either mutant *FMR1* RNAs or an ASO that disturbed the structure of the hairpin formed by CGGs in *FMR1* RNA. In the first experiment, we designed two mutant constructs with either extended (Ins18nt) or shortened (Del15nt) distances between the ACG (+1) near-cognate start codon and the hairpin formed by CGG repeats (Fig. 5A and Supplementary Fig. S5a and d). To provide a more physiological context for CGG repeat length, in addition to constructs containing 16 and 85 CGG repeats, we also generated plasmid constructs with 29 CGG repeats containing two interruptions, which fall within the range typically observed in the healthy population. As expected, the level of FMRpolyG-Nluc-FLAG was significantly lowered for all Ins18 nt mutant RNAs: with 16 CGGs (Fig. 5B and Supplementary Fig. S5b), 29 CGGs (Fig. 5C), and 85 CGG repeats (Fig. 5D). For all lengths of CGG tracts, this effect resulted entirely from impaired translation as mRNA levels remained unchanged or slightly increased (Supplementary Fig. S5c). Surprisingly, shortening the distance between ACG (+1) and CGGs in Del15nt constructs resulted in a significant increase in the FMRpolyG-Nluc-FLAG levels for all tested CGG repeat lengths (Fig. 5B). However, analysis of mRNA levels showed that this increase was partially driven by elevated transcript abundance (Supplementary Fig. S5c). Together, these results indicate that a non-optimal distance between the ACG (+1) codon and the CGG repeats negatively affects FMRpolyG-Nluc-FLAG translation initiation.

**Figure 6.**
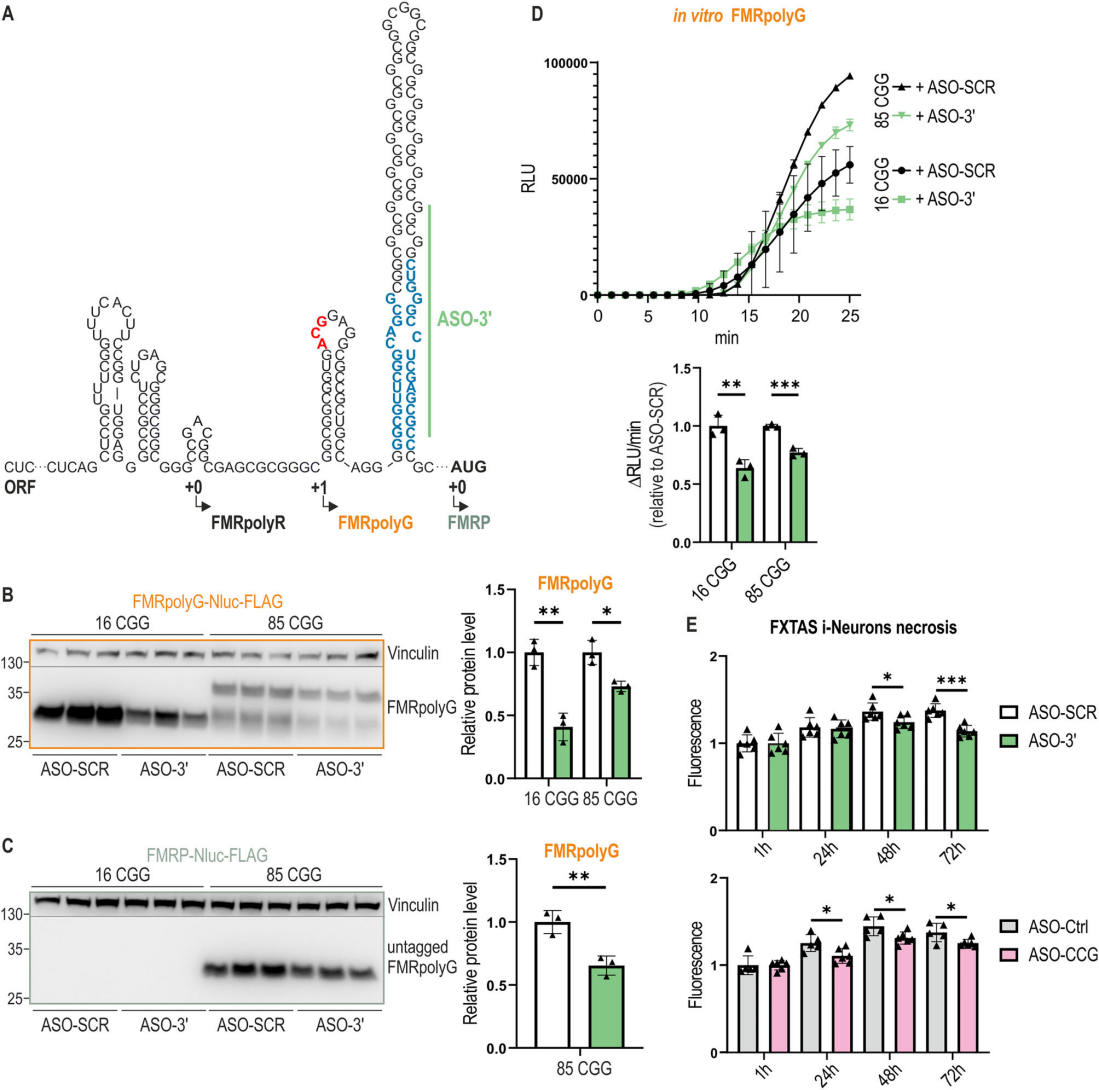
ASO-3′ targeting the stabilizing region of the CGG-derived hairpin within the *FMR1* 5′UTR lowers FMRpolyG levels and reduces necrosis in FXTAS iPSC-derived neurons. (**A**) The predicted RNA secondary structure of *FMR1* 5′UTR containing 16 CGGs with marked target sequence for binding of ASO-3′. The flanking regions involved in CGG-hairpin stabilization are marked in blue. (**B**) WB analysis and corresponding quantification of FMRpolyG-Nluc-FLAG levels in HEK-293 cells 48 h post-transfection with WT construct with 16 or 85 CGGs and administration of 200 nM of either control ASO-SCR or ASO-3′ oligonucleotides (3 h after plasmid delivery). (**C**) The same as in panel (B), but using FMRP-Nluc-FLAG construct with 16 and 85 CGGs, from which the untagged FMRpolyG protein containing a homomeric 85 glycine tract is translated. In panels (B) and (C), results for FMRpolyG were normalized to Vinculin. A specific anti-FMRpolyG antibody was used to detect both FMRpolyG-Nluc-FLAG (B) and untagged FMRpolyG (C). The graphs represent averages for *N* = 3 biologically independent samples with SDs relative to WT = 1. (**D**) The *FMR1* mRNAs with ACG (+1) near-cognate start codons containing either 16 or 85 CGG repeats were *in vitro* transcribed and used as templates for *in vitro* translation in the presence of 100 nM control ASO-SCR or the *FMR1*-targeting ASO-3′. Relationship between time and the luminescence signal from enzymatic reaction catalysed by FMRpolyG-Nluc *in vitro* translated from indicated reporter mRNAs is shown (left; RLU, relative luminescence units). Quantification of the effect of the ASO-3′ on the translation efficiency of *FMR1* mRNAs with 16 or 85 CGGs (right; ΔRLU/min—the change of the luminescence signal over time). *N* = 3 independent experiments. (**E**) Treatment with ASO-3′ (upper panel) or ASO-CCG (lower panel) reduces necrosis in FXTAS (72 CGG) iPSC-derived neurons. Necrosis was quantified as fluorescence intensity (relative fluorescence units). The graphs represent averages for *N* = 5 biologically independent samples with SDs relative to 1; Statistical analysis was performed using multiple unpaired parametric *t*-tests with Holm–Šídák correction for multiple comparisons or an unpaired two-sided *t*-test; *P < .05; **P < .01; ***P < .001, ns, non-significant.

ASO blockers can sterically hinder initiation from the targeted start codon for the RAN translation of *FMR1* mRNA if they are complementary to the region of ACG +1 [37]. Moreover, we previously showed that ASOs directly targeting CGGexp within the *FMR1* 5′UTR (ASO-CCG) slow down ribosome movements on mRNA and reduce the level of FMRpolyG [53]. The ASOs targeting regulatory elements and uORFs to modulate the level of the protein under study were also successfully used in other studies [54, 55]. Therefore, we decided to test how the initiation of RAN translation would be regulated by the binding of a 20-nt-long ASO to the 3′ flanking sequence involved in the formation of CGG hairpin (ASO-3′) (Fig. 6A). Since this region is likely responsible for the stabilization of CGG hairpin [44], we assumed that ASO could significantly affect both the stability of this RNA structure and the distance of the ACG +1 near-cognate start codon and CGG hairpin (Supplementary Fig. S6a).

Treatment of HEK-293 cells expressing WT constructs with 16 or 85 CGGs in the +1 frame with ASO-3′ resulted in a significant depletion in FMRpolyG-Nluc-FLAG synthesis from both model RNAs with short and long CGG repeats (Fig. 6B). The effect of ASO-3′ suggests that the result was independent of the number of CGG repeats, however, it was partially associated with a reduction in mRNA levels (Supplementary Fig. S6b). Importantly, we also observed a decrease in untagged FMRpolyG translated from ACG (+1) initiated CGG repeats in the FMRP-Nluc-FLAG construct following ASO-3′ treatment (Fig. 6C), while the corresponding mRNA levels remained unchanged (Supplementary Fig. S6b). As ASO targets the CGG hairpin stabilizing region on the 3′ site, this interaction may lead to the unwinding of the hairpin base. Therefore, the possibly extended distance between the ACG (+1) near-cognate start codon and the hairpin formed by CGG repeats could be the reason for the observed results.

To verify whether changes in the FMRpolyG-Nluc-FLAG level resulted directly from ASO-3′ binding, we performed *in vitro* translation with an equimolar amount of RNAs *in vitro* transcribed from appropriate DNA templates. We used cell-free rabbit reticulocyte lysate to translate *in vitro* RNA encoding the 5′UTR sequence of the *FMR1* gene with a native ACG (+1) near-cognate start codon and either 16 or 85 CGG repeats in the presence of ASO-SCR or ASO-3′. Similar to transient expression in the cellular system, also in these *in vitro* experiments, we observed a strong decrease in the level of FMRpolyG protein that was produced (Fig. 6D).

To highlight the therapeutic relevance of our results, we delivered ASO-3′ to FXTAS iPSC-derived neurons (i-Neurons) to investigate the effect of the applied ASO on patient cell necrosis. As a positive control, we used short ASOs composed exclusively of LNA units—ASO-CCG—targeting directly CGG repeats and corresponding ASO-Ctrl (previously described in [53]. We found that ASO-3′ treatment, similar to ASO-CCG, mitigated necrosis in FXTAS iPSC-derived neurons (Fig. 6E).

These results showed that the optimal distance between the ACG (+1) codon and downstream RNA structure formed by CGG repeats has a considerable effect on the initiation of FMRpolyG synthesis. In addition, this process can, to some extent, be negatively regulated by the steric obstacle located in a non-optimal distance downstream of the start codon, which can decrease the dwell time of the scanning PIC at the near-cognate codon. Consistent with this, ASO-3′ targeting the structure formed by CGG repeats significantly reduces the toxicity evoked by RNA containing expanded CGG repeats.

### Translation initiation and elongation of different reading frames of *FMR1* mRNA is CGG repeat length-dependent

The *FMR1* mRNA level has been shown to increase in the PM range as CGG repeats expand [13, 14, 16, 18]. However, the increase in the number of CGG repeats was also correlated with the gradual impairment of canonical *FMR1* translation efficiency and reduced level of FMRP [14, 17–19]. While the level of FMRpolyG protein may depend on several factors, the number of CGG repeats appears to be one of the primary and most important factor regulating the translation of *FMR1* mRNA.

**Figure 7.**
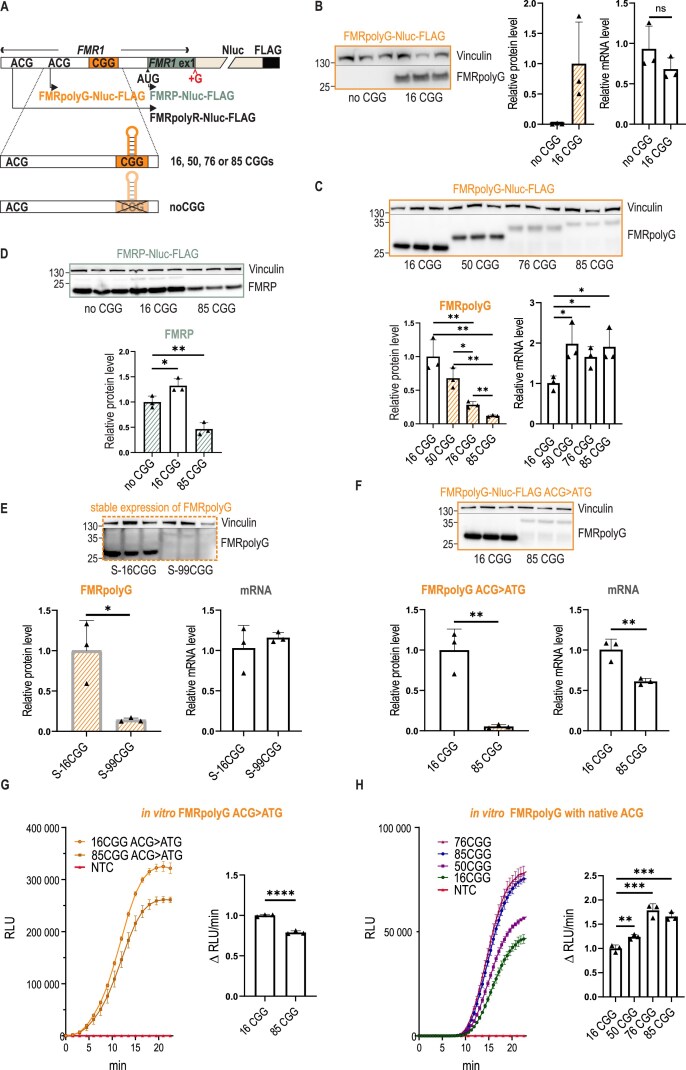
The efficiency of *FMR1* translation is influenced by the size of CGG repeats. (**A**) Scheme of cloned constructs with different number of CGG repeats. (**B, C**) FMRpolyG-Nluc-FLAG level depends on the CGG repeats’ size in encoding mRNAs. WB analyses and corresponding quantifications of FMRpolyG-Nluc-FLAG level in HEK-293 cells 24 h post-transfection with constructs harboring indicated number of CGGs. Graphs represent averages for *N* = 3 biologically independent samples with SDs, normalized to Vinculin. The effect of different number of CGG repeats on the mRNA level quantified by RT-qPCR and normalized to GAPDH (B, C; right graphs). (**D**) The level of FMRP-Nluc-FLAG depends on the CGG repeats’ size in encoding mRNAs. WB analysis and corresponding quantification of FMRP-Nluc-FLAG level in HEK-293 cells 24 h post-transfection with constructs harboring indicated number of CGGs. (**E**) WB analysis and corresponding quantification of RAN-translated FMRpolyG levels containing 16 or 99 CGGs in two cell models with stable expression of FMRpolyG upon 7 days of doxycycline induction (left panel). The level of S-16CGG and S99-CGG transgenes’ expression was quantified with RT-qPCR and normalized to GAPDH (right panel). (**F**) Changes in the efficiency of translation of FMRpolyG-Nluc-FLAG initiated from mutant ACG > AUG codon in mRNA with 16 or 85 CGGs. WB analysis and corresponding quantification of FMRpolyG-Nluc-FLAG level in HEK-293 cells 24 h post-transfection with indicated constructs (left panel). The effect of introduced ACG > AUG mutation on the mRNA level was quantified by RT-qPCR and normalized to GAPDH (right panel). In panels (B–F), results were normalized to Vinculin. The graphs represent averages for *N* = 3 biologically independent samples with SDs, relative to 16 CGG = 1 (B, C), no CGG = 1 (D), S-16CGG = 1 (E), and ACG > ATG mutant with 16 CGGs = 1 (F). (**G**) The effect of 16 and 85 CGGs on the translation elongation during *in vitro* translation of *FMR1* mRNAs in +1 frame with ACG > ATG mutation. The *FMR1* mRNAs in +1 frame with mutant ACG > AUG codon containing different number of CGG repeats were *in vitro* transcribed and used as template for *in vitro* translation. *N* = 3 independent experiments. The relationship between reaction time and luminescence for *in vitro* translation assay of indicated Nluc reporter mRNAs is presented. The values on the graphs are presented relative to 16 CGG ACG > ATG = 1. (**H**) The effect of the different number of CGG repeats on the efficiency of both translation initiation and elongation of *FMR1* mRNAs in +1 frame with native ACG (+1) near-cognate start codon during *in vitro* translation. The *FMR1* mRNAs in +1 frame with ACG (+1) native near-cognate start codon containing different number of CGG repeats were *in vitro* transcribed and used as template for *in vitro* translation. *N* = 3 independent experiments. The relationship between reaction time and luminescence for *in vitro* translation assay of indicated Nluc reporter mRNAs is presented. The values on the graphs are presented relative to 16 CGG ACG = 1. NTC, no template control; RLU, relative luminescence units; ΔRLU/min, the change of the luminescence signal over time. Statistical analysis was performed using multiple unpaired parametric *t*-tests with Holm–Šídák correction for multiple comparisons or an unpaired two-sided *t*-test; *P < .05; **P < .01; ***P < .001, ****P < .0001, ns, non-significant.

To verify how different lengths of CGG repeats affect *FMR1* translation, we cloned constructs containing different numbers of CGG repeats (16, 50, 76, and 85) or those possessing no repeats (noCGG) (Fig. 7A and Supplementary Fig. S7a). A lack of CGG repeats led to a near-complete loss of FMRpolyG-Nluc-FLAG initiated at the ACG (+1) codon, which was confirmed by both anti-FMRpolyG antibody (characterized by reduced sensitivity due to an incomplete epitope in the mutant protein; Fig. 7B; left panel) and anti-FLAG antibody (Supplementary Fig. S7b). Of note, the mRNA level remained unaffected (Fig. 7B; right panel). This result confirms that the translation initiation of FMRpolyG at the ACG (+1) codon requires the structural obstacle formed by CGG repeats.

For constructs with different numbers of CGG repeats, we observed a repeat-length-dependent decrease in the FMRpolyG-Nluc-FLAG protein level (Fig. 7C; left panel) with a simultaneous increase (up to 2 times) in the mRNA level (Fig. 7C; right panel). This indicates that differences in the FMRpolyG-Nluc-FLAG level in the designed model resulted from translation changes. Of note, mRNA with 85 CGGs resulted in depletion of the FMRP-Nluc-FLAG level (Fig. 7D), suggesting that both FMRpolyG and FMRP translations are regulated by CGG repeat tracts.

We also analysed the level of FMRpolyG in HEK-293 FlipIn T-Rex cell models with a stable expression of FMRpolyG from mRNA containing either 16 or 99 CGG repeats (S-16CGG and S-99CGG, respectively) [38]. Similar to the transient expression system, we observed a strong reduction in FMRpolyG level produced from the transgene with longer repeats (Fig. 7E; left panel), which was not influenced by the mRNA level (Fig. 7E; right panel). Together, these data confirm that increases in the number of CGG repeats are correlated with the gradual impairment of the FMRpolyG steady-state level.

Bearing in mind that the steady-state level of a protein is a representation of various processes and mechanisms, including the mRNA level, the efficiency of translation initiation and elongation, and protein aggregation and its stability, we wanted to separate the effect of particular mechanisms on the observed FMRpolyG level. To distinguish the effect of long CGGs on translation initiation and elongation, we forced the equal efficiency of translation initiation by using constructs containing an ACG > ATG mutation with either 16 or 85 CGGs. We showed that longer CGG repeats significantly decreased the level of FMRpolyG-Nluc-FLAG (Fig. 7F; left panel, and Supplementary Fig. S7c). Surprisingly, we also observed a significant decrease in the mRNA level (Fig. 7F; right panel), suggesting that disturbed elongation of FMRpolyG translation may decrease mRNA stability.

To exclude other factors regulating mRNA stability, protein aggregation and potentially limiting amount of aminoacyl-tRNAs in the cell, we performed an *in vitro* translation of the 5′UTR sequence of the *FMR1* gene with the ACG > ATG mutation (to ensure the same efficiency of translation initiation) and either 16 or 85 CGG repeats. Following data from the transient expression system, the *in vitro* translation confirmed the decreased efficiency of translation elongation from mRNA with longer CGG repeats (Fig. 7G). However, the much lower protein depletion upon the *in vitro* condition suggests that the observed decrease in the FMRpolyG-Nluc-FLAG level in cells resulted also from other mechanisms. Alternatively, other factors regulating the translation of FMRpolyG may be at play *in cellulo*.

To verify how RNA secondary structures formed by different numbers of CGG repeats will affect the process of translation initiation at the ACG (+1) codon (which is much more sensitive to PIC pausing than the AUG codon), we performed similar *in vitro* translations with RNAs encoding the native 5′UTR sequence of the *FMR1* gene with 16, 50, 76 and 85 CGGs (both translation initiation and elongation were considered). We observed a significant repeat-length dependent increase in the level of produced FMRpolyG protein, suggesting that the translation initiation at the ACG (+1) near-cognate start codon is enhanced by more stable CGG-derived secondary structures and outweighs the inhibitory effect on elongation (Fig. 7H).

We concluded that both +0 and +1 frames of *FMR1* mRNA are CGG repeat-length dependent. Initiation of FMRpolyG translation at the ACG (+1) near-cognate start codon is, to some extent, positively regulated by the increasing number of CGG repeats within the 5′UTR of *FMR1* mRNA; however, this positive effect is completely invisible in cellular conditions. Importantly, as the efficiency of translation elongation is negatively correlated with expanding CGGs during *in vitro* translation, this effect may be enhanced *in cellulo* and, therefore, contributes to the observed decrease in the FMRpolyG steady-state level.

### Native FMRpolyG translated from *FMR1* containing short CGG repeats is rapidly degraded by proteasome

**Figure 8.**
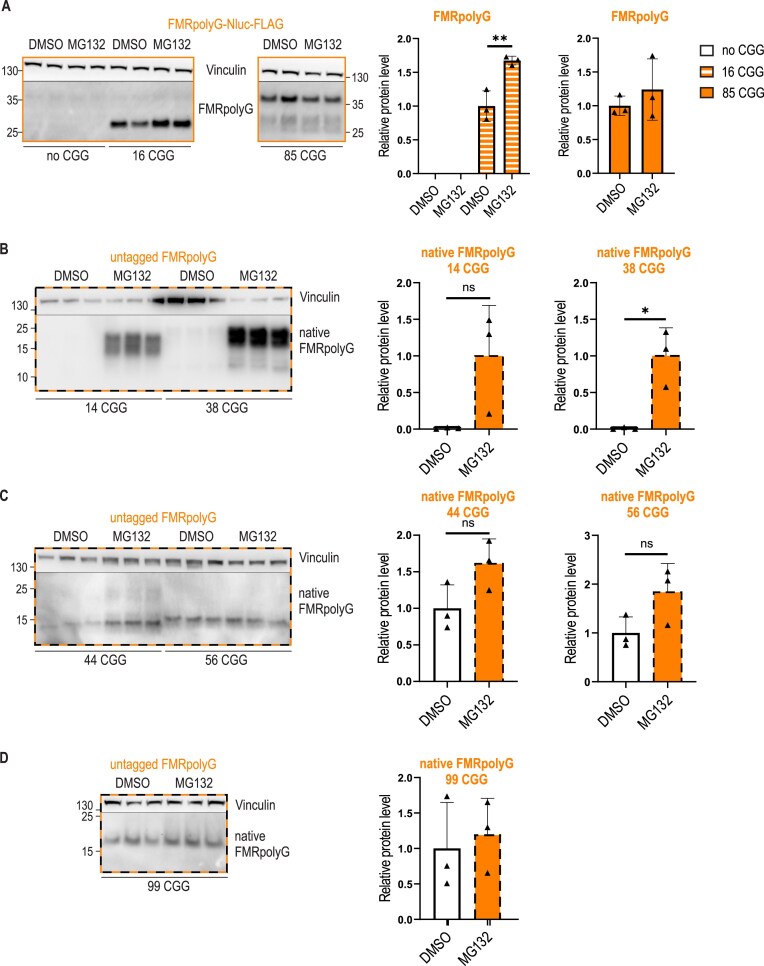
The stability of FMRpolyG protein depends on the CGG repeat length in coding mRNA. (**A**) WB analysis and corresponding quantification of tagged FMRpolyG-Nluc-FLAG level in HEK-293 cells transfected with WT constructs in +1 frame containing different numbers of CGGs followed by 6-h treatment with either 5 µM MG132 proteasome inhibitor or DMSO used as a control. (**B**–**D**) WB analysis and corresponding quantification of native, untagged FMRpolyG level in HEK-293 cells transfected with constructs encoding 5′UTR *FMR1* in +1 frame without any tag followed by treatment with 5 µM MG132 or DMSO control treatment for 6 h. Results were normalized to Vinculin. Graphs represent averages for *N* = 3 biologically independent samples with SDs relative to DMSO treated samples = 1 (A, C, D) and MG132 treated samples = 1 (B); Statistical analysis was performed using an unpaired two-sided *t*-test; *P < .05; **P < .01, ns, non-significant.

Depending on the fusion partner, the threshold of FMRpolyG detection varies [34]. This observation is in line with the fact that small peptides translated from uORFs are usually barely detectable, and fusion with large tags leads to increased cellular stability and easier detection using WB [56]. In consequence, differences in the level of FMRpolyG detection may result from the effect of the tag used on protein stability; thus, the differences in the efficiency of RAN translation may be underestimated. Therefore, to study the potential effect of fusion with the Nluc-FLAG on FMRpolyG stability, we performed experiments where plasmids encoding the 5′UTR of *FMR1* with no CGGs, 16 or 85 CGGs were delivered to HEK-293 cells followed by proteasome inhibition (Fig. 8A and Supplementary Fig. S8a). Treatment with an MG132 inhibitor stopped the proteasome-dependent pathway of protein degradation and allowed us to monitor short-lived proteins. The WB analysis showed that (as observed in previous experiments) loss of FMRpolyG-Nluc-FLAG upon the noCGG mutation did not affect protein turn-over as no signal was detected upon proteasome inhibition (Fig. 8A). We observed an ~1.5-fold increase of FMRpolyG-Nluc-FLAG with 16 CGGs upon MG132 treatment and no change in the level of FMRpolyG-Nluc-FLAG protein containing 85 CGG repeats, suggesting that FMRpolyG in fusion with the Nluc-FLAG are generally quite stable—especially ones with a long polyglycine tract.

FMRpolyG translated from the normal range of CGGs has not been detected in human samples [25, 34]. This may result from the extremely short half-life of native FMRpolyG. However, from mutant *FMR1* mRNA with expanded CGGs, FMRpolyG can be detected by either WB or immunohistochemical analyses. To verify whether CGG repeat length can influence FMRpolyG stability, we delivered plasmids encoding the 5′UTR of the *FMR1* gene with 14, 24, 38, 44, 56, or 99 CGG repeats, without any tag, to HEK-293 cells and proceeded with the MG132 treatment (Fig. 8B–D and Supplementary Fig. S8b). We found that untagged FMRpolyG produced from mRNA with longer CGGs (44, 56, and 99) was easily detectable via WB in both the control and MG132-treated samples (Fig. 8C–D). However, the untagged FMRpolyG from mRNA containing shorter CGGs (14, 24, and 38) was detected exclusively upon proteasome inhibition (Fig. 8B and Supplementary Fig. S8b). Similar results were obtained upon another proteasome inhibitor treatment—Bortezomib (Supplementary Fig. S8c–d).

This finding shows that native FMRpolyG with a short polyglycine tract is synthesized but characterized by a very short half-life, which makes this protein difficult to detect. However, native FMRpolyG containing a long polyglycine tract is resistant to proteasome activity with a threshold of 40–50 CGG repeats..

## Discussion

Long polyglycine tract-containing FMRpolyG protein is synthesized through RAN translation from mutant *FMR1* mRNA containing expanded CGG repeats. It is one of the pathogenic factors involved in the aetiology of FXPAC. RAN translation products are prone to accumulate in ubiquitin-positive intranuclear or perinuclear aggregates. While the RAN translation of *FMR1* was first described a decade ago [26], the precise mechanism of this non-canonical process is not fully understood.

The efficiency of translation initiation from near-cognate start codons is significantly lower than from the canonical one. We showed that the single nucleotide substitution of ACG (+1) to the canonical AUG start codon increases the efficiency of FMRpolyG-Nluc-FLAG biosynthesis by 10–30 times (Fig. 2B and Supplementary Fig. S2b). Interestingly, mutations to other near-cognates (e.g. CUG and GUG) had a marginal effect. These results suggest that only a small fraction of scanning 43S PICs pause at ACG (+1) (which initiates the assembling of the 80S ribosome), while most PICs scan further to the AUG codon that initiates FMRP translation. Of note, the ACG (+1) near-cognate codon is well conserved across different species [37] suggesting that its position and local sequence context is required for FMRpolyG RAN translation regulation. Further, our results emphasize that the sequence and structural context of the near-cognate ACG (+1) codon have a pivotal role in the efficiency of both RAN and canonical translations of normal and mutant *FMR1* mRNAs.

In the 1980s, Marylin Kozak performed landmark experiments providing the first suggestions concerning how sequences and secondary structures in the vicinity of the start codon influence translation initiation [47, 57]. It was established that the specific nucleotide positions surrounding the AUG start codon strongly affected the efficiency of translation initiation across various vertebrates’ mRNAs [47, 58, 59]. Importantly, these surrounding nucleotides had a larger influence on the recognition of non-AUG start codons than on the AUG start codons [46, 60]. Our experiments on the strength of the Kozak sequence context demonstrated that the sequence surrounding the ACG (+1) near-cognate start codon is crucial for the efficient RAN translation initiation of FMRpolyG-Nluc-FLAG (Fig. 3), as a non-optimal Kozak sequence context dramatically reduced the initiation of FMRpolyG-Nluc-FLAG translation at the ACG (+1) near-cognate start codon.

According to the scanning model of translation initiation, it was shown that mRNA structures located either upstream or downstream of the start codons can influence the efficiency of initiation by regulating the dynamics of PIC movement [51]. Therefore, initiation from the near-cognate start codon or the AUG start codon located in the poor context can be increased when a stable RNA secondary structure is placed downstream from the start codon [50, 61]. It is worth mentioning that the distance between the start codon and the secondary structure is important since it directly results in the positioning of stalled PIC on the mRNA. Both normal and expanded CGGs within the 5′UTR of *FMR1* mRNA can form thermodynamically stable hairpin structures [62]. Expanded repeats were also confirmed to be involved in G4–RNA-quadruplex formation [63, 64]. Because of this, the dynamics of ribosomes can be disturbed at the initiation and elongation steps. Previously, it has been shown that inhibition of the RNA helicases, eIF4A or DHX15—which are involved in ribosome scanning—abolished the RAN translation of expanded CGG repeats [27, 38]. The involvement of secondary structures in the modulation of RAN translation has also been confirmed for another RED caused by the expansion of GGGGCC repeats (G4C2) within the *C9orf72* gene that forms in very stable G4–RNA-quadruplex structures [65, 66]. A recent genome-wide analysis (which is in agreement with these results) revealed that secondary structures located downstream of functional near-cognate start codons are a common feature of mRNAs [67, 68].

Our results show that the presence and positioning of thermodynamically stable secondary RNA structures within the 5′UTR of *FMR1* mRNA has a substantial impact on the efficiency of RAN translation initiation and is significantly stronger than the optimal sequence context of the near-cognate start codon. Depending on the distance between the near-cognate start codon and the stable hairpin structure, the initiation can be either significantly enhanced or silenced [50]. A distance of 2 and 6 nt has a strongly negative effect on FMRpolyG-Nluc-FLAG translation initiation (Fig. 4E), while extending this distance to 14 and 20 nt results in an extremely high increase in the FMRpolyG-Nluc-FLAG level. We observed similar relationships when the ACG (+1) was moved farther away or closer to a stable structure formed by the CGGs (Fig. 5B–D). These results suggest that near-cognate codons can only be used as efficient start codons if located in an optimal structural context that slows down or pauses PIC scanning.

Here, we show that targeting the 3′ stabilizing region of the hairpin formed by CGG repeats reduces FMRpolyG-Nluc-FLAG levels from *FMR1* mRNAs containing both short and long CGG tracts (Fig. 6B). Although ASO-3′ treatment led to a modest decrease in mRNA levels (Supplementary Fig. S6b), the observed effect was also associated with impaired translation. This conclusion is supported by the reduction in untagged FMRpolyG translated from expanded CGG repeats of the FMRP-Nluc-FLAG construct (Fig. 6C), where mRNA levels remained unchanged (Supplementary Fig. S6b). Furthermore, translation impairment was confirmed in more controlled *in vitro* experiments in which the contribution of mRNA stability was eliminated (Fig. 6D). Finally, we found that ASO-3′ treatment reduced necrosis in FXTAS iPSC-derived neurons (Fig. 6E), suggesting that toxicity associated with the expression of expanded CGG repeat-containing RNA was alleviated.

These findings suggest that targeting the structure formed by CGG repeats with ASO blocker is a promising therapeutic strategy; however, several challenges must be addressed, including efficient delivery, potential off-target effects, and toxicity, as well as the optimal timing of administration. This is particularly relevant in FXTAS and other neurodegenerative disorders, where ASO treatment would likely need to be initiated prior to symptom onset, requiring early identification of at-risk individuals. However, given that not all carriers of the *FMR1* PM develop FXTAS, a deeper understanding of the molecular mechanisms underlying disease etiology is required to guide such strategies. Remarkably, the successful therapies such as Nusinersen and Eteplirsen, developed for spinal muscular atrophy and Duchenne muscular dystrophy, respectively, provide a strong rationale for further exploration of ASO-based approaches and support the continued development of gene-targeted therapeutics.

The structures formed by expanded CGG repeats are predicted to be extremely stable. Therefore, they may form a physical obstacle for scanning PICs or an elongating ribosome. Here, we showed that indeed RAN translation was strongly dependent on the presence of CGG repeats, as barely any RAN protein was produced upon the deletion of CGGs (Fig. 7B and Supplementary Fig. S7b). However, depending on the stage of translation, the CGG repeat length may have either a positive or negative effect. We observed that mRNAs containing a native ACG (+1) near-cognate start codon were more efficiently translated *in vitro* as CGG repeats increased (Fig. 7H). These data confirm that longer CGGs positively regulate the initiation at ACG (+1). It is noteworthy that non-AUG initiation is exceptionally sensitive to conditions that slow down or pause the progression of 43S ribosome scanning. In line with this, it has been shown that when translation elongation is inhibited by cycloheximide, the level of non-AUG translation is elevated [69], which may directly result from the queuing of PICs and/or 80S ribosomes. In contrast, expanding CGG repeats are negatively correlated with the efficiency of FMRpolyG translation elongation (Fig. 7G).

Our cell-based assays presented a repeat length-dependent decrease in the steady-state level of FMRpolyG-Nluc-FLAG upon CGG expansion (Fig. 7C). The significantly lower levels of long FMRpolyG-Nluc-FLAG produced from mRNA containing either ACG (+1) or mutant AUG as the initiation codon suggests that the observed decrease in protein levels primarily resulted from impaired translation, which was confirmed by *in vitro* experiments (Fig. 7G–H). The observed discrepancies concerning the FMRpolyG level upon expansion of CGG repeats between *in vitro* and *in cellulo* conditions suggest that other mechanisms affecting the steady-state protein level may be at play in cells (e.g. differences in localization or stability of mRNA in the cell, different efficiency of translation elongation, and different stability or aggregation of short and long polyglycine tract-containing proteins).

Previously, it was shown that the increase in the number of CGG repeats within *FMR1* mRNA was correlated with decreasing efficiency of the canonical translation of FMRP [14, 17–19, 37]. Intriguingly, the GC-rich character and highly structured *FMR1* 5′UTR with expanded CGG repeats should theoretically inhibit translation since the formation of a strong secondary structure within the 5′UTR has been reported to inhibit ribosomal scanning [57, 70, 71]. Nevertheless, FXTAS patients present only slightly reduced levels of FMRP and increased levels of *FMR1*, suggesting that an increased transcription rate or elevated mRNA stability may constitute a compensatory effect to lowered FMRP levels. Here, we showed that the translation in the FMRpolyG frame from the ACG (+1) near-cognate start codon had only a marginal influence on the FMRP frame (Fig. 2B; ACG > AAA mutation). However, the mutation of the ACG > AUG codon, according to the first-AUG rule [72, 73], resulted in a robust increase in the level of FMRpolyG-Nluc-FLAG protein and abolished translation of the FMRP-Nluc-FLAG. The loss of the FMRP-Nluc-FLAG resulted from the fact that the uORF of FMRpolyG was translated at the efficiently utilized AUG codon; hence, hardly any ribosomes were able to initiate translation at the downstream AUG start codon of FMRP. Of note, we observed nearly a three-fold decrease in the level of FMRP from transcripts containing 85 CGGs compared to 16 CGG repeats (Fig. 7D), which may have resulted from the ineffective scanning through expanded CGGs. This observation is also supported by the results from mutants with artificial hairpin structures as [independent of the distance between the ACG (+1) near-cognate start codon and the hairpin structure], the FMRP is barely detectable (Fig. 4E).

Sellier and co-workers demonstrated that depending on the fusion partner, the threshold of FMRpolyG detection varies [34]. Importantly, there was a difference in the correlation between the FMRpolyG level and the number of CGG repeats that depended on the fusion partner. In other words, when FMRpolyG-FLAG was used, a positive correlation between the level of FMRpolyG and CGG size was observed. However, when FMRpolyG was fused to GFP, no correlation was observed. Nluc, which we used in this study, is a smaller tag than GFP and should not affect the stability of the fused protein. Accordingly, only a slight positive effect of proteasome inhibition was observed on the stability of FMRpolyG-Nluc-FLAG containing a short polyglycine tract, while the stability of proteins encoded by expanded CGGs was unchanged (Fig. 8A).

RAN translation has also been confirmed to occur at mRNA containing a normal range of CGG repeats in the reporter system [37] but has never been detected in patient samples. Here, we showed that untagged FMRpolyG proteins produced from mRNA with short CGG repeats (14, 24, and 38) were undetectable due to extremely short protein half-live caused by quick proteasome-induced degradation (Fig. 8B and Supplementary Fig. S8b and c). This result confirms that FMRpolyG can be translated from mRNA with a normal range of CGG repeats. As FMRpolyG proteins containing 44, 56, and 99 CGG repeats were readily detectable irrespective of MG132 treatment, these data suggest the existence of a threshold of ~40 CGG repeats that determines FMRpolyG stability. In addition, as mutated FMRpolyG co-localizes with ubiquitin-positive intranuclear inclusions in FXTAS patients, our data suggest that toxic FMRpolyG can be ubiquitinated at least in aggregates but degraded by proteasomes with much lower efficiency compared to short polyglycine tract-containing FMRpolyGs.

In sum, the regulation of the RAN translation of *FMR1* mRNA is an extremely complex, multilayered and elusive process. There is an interplay between the sequence and structure formed within the 5′UTR that jointly modulates the efficiency of FMRpolyG and FMRP translation. Nevertheless, the comprehensive analysis of structural dependencies affecting RAN translation presented in this work suggests that the regulation of translation initiation that depends on secondary RNA structures may have a universal character and be reflected in other REDs. Moreover, RNA structures with expanded repeats may serve as druggable targets.

## Supplementary Material

gkag569_Supplemental_File

## Data Availability

The data underlying this article are available in Zenodo at https://doi.org/10.5281/zenodo.20134621.

## References

[1] Malik I., Kelley C.P., Wang E.T et al. Molecular mechanisms underlying nucleotide repeat expansion disorders. Nat Rev Mol Cell Biol. 2021;22:589–607. 34140671 10.1038/s41580-021-00382-6 PMC9612635

[2] Depienne C., Mandel J.L. 30 years of repeat expansion disorders: what have we learned and what are the remaining challenges?. Am J Hum Genet. 2021;108:764–85. 10.1016/j.ajhg.2021.03.011 33811808 PMC8205997

[3] Darnell J.C., Richter J.D. Cytoplasmic RNA-binding proteins and the control of complex brain function. Cold Spring Harb Perspect Biol. 2012;4:1–17. 10.1101/cshperspect.a012344 PMC3405866 22723494

[4] Sidorov M.S., Auerbach B.D., Bear M.F. Fragile X mental retardation protein and synaptic plasticity. Mol Brain. 2013;6:1–11. 10.1186/1756-6606-6-15 23566911 PMC3636002

[5] Kunst C.B., Warren S.T. Cryptic and polar variation of the fragile X repeat could result in predisposing normal alleles. Cell. 1994;77:853–61. 10.1016/0092-8674(94)90134-1 7911740

[6] Broniarek I., Niewiadomska D., Sobczak K. Contribution of DNA/RNA structures formed by expanded CGG/CCG repeats within the FMR1 locus in the pathogenesis of fragile X-associated disorders. Wiley Interdiscip Rev RNA. 2024;15:e1874. 39523485 10.1002/wrna.1874

[7] Hagerman R.J., Hagerman P. Advances in clinical and molecular understanding of the FMR1 premutation and fragile X-associated tremor/ataxia syndrome. Lancet Neurol. 2013;12:786–98. 10.1016/S1474-4422(13)70125-X 23867198 PMC3922535

[8] Hagerman R.J., Hagerman P. Fragile X-associated tremor/ataxia syndrome-features, mechanisms and management. Nat Rev Neurol. 2016;12:403–12. 10.1038/nrneurol.2016.82 27340021

[9] Allen E.G., Charen K., Hipp H.S et al. Refining the risk for fragile X-associated primary ovarian insufficiency (FXPOI) by FMR1 CGG repeat size. Genet Med. 2021;23:1648–55. 10.1038/s41436-021-01177-y 33927378 PMC8460441

[10] Rosario R., Stewart H.L., Choudhury N.R et al. Evidence for a fragile X messenger ribonucleoprotein 1 (FMR1) mRNA gain-of-function toxicity mechanism contributing to the pathogenesis of fragile X-associated premature ovarian insufficiency. FASEB J. 2022;36:1–17. 10.1096/fj.202200468RR PMC9828574 36250920

[11] Hagerman R.J., Protic D., Rajaratnam A et al. Fragile X-associated neuropsychiatric disorders (FXAND). Front Psychatry. 2018;9:1–9. 10.3389/fpsyt.2018.00564 PMC6243096 30483160

[12] Tassone F., Hagerman R.J., Chamberlain W.D et al. Transcription of the FMR1 gene in individuals with fragile X syndrome. Am J Med Genet. 2000;97:195–203. 10.1002/1096-8628(200023)97:3<195::AID-AJMG1037>3.0.CO;2-R 11449488

[13] Tassone F., Hagerman R.J., Taylor A.K et al. Elevated levels of FMR1 mRNA carrier males: a new mechanism of involvement in the fragile-X syndrome. Am J Hum Genet. 2000;66:6–15. 10.1086/302720 10631132 PMC1288349

[14] Primerano B., Tassone F., Hagerman R.J et al. Reduced FMR1 mRNA translation efficiency in fragile X patients with premutations. RNA. 2002;8:1482–8. 10.1017/S1355838202020642 12515381 PMC1370354

[15] Kenneson A., Zhang F., Hagedorn C.H et al. Reduced FMRP and increased FMR1 transcription is proportionally associated with CGG repeat number in intermediate-length and premutation carriers. Hum Mol Genet. 2001;10:1449–54. 10.1093/hmg/10.14.1449 11448936

[16] Dias C.M., Issac B., Sun L et al. Glial dysregulation in the human brain in fragile X-associated tremor/ataxia syndrome. Proc Natl Acad Sci USA. 2023;120:e2300052120. 37252957 10.1073/pnas.2300052120 PMC10265985

[17] Feng Y., Zhang F., Lokey L.K et al. Translational suppression by trinucleotide repeat expansion at FMR1. Science (1979). 1995;268:731–4. 10.1126/science.7732383 7732383

[18] Chen L.S., Tassone F., Sahota P et al. The (CGG)n repeat element within the 5′ untranslated region of the FMR1 message provides both positive and negative *cis* effects on *in vivo* translation of a downstream reporter. Hum Mol Genet. 2003;12:3067–74. 10.1093/hmg/ddg331 14519687

[19] Ludwig A.L., Espinal G.M., Pretto D.I et al. CNS expression of murine fragile X protein (FMRP) as a function of CGG-repeat size. Hum Mol Genet. 2014;23:3228–38. 10.1093/hmg/ddu032 24463622 PMC4030777

[20] Tassone F., Beilina A., Carosi C et al. Elevated FMR1 mRNA in premutation carriers is due to increased transcription. RNA. 2007;13:555–62. 10.1261/rna.280807 17283214 PMC1831862

[21] Sellier C., Rau F., Liu Y et al. Sam68 sequestration and partial loss of function are associated with splicing alterations in FXTAS patients. EMBO J. 2010;29:1248–61. 10.1038/emboj.2010.21 20186122 PMC2857464

[22] Qurashi A., Li W., Zhou J.Y et al. Nuclear accumulation of stress response mRNAs contributes to the neurodegeneration caused by fragile X premutation rCGG repeats. PLoS Genet. 2011;7:e1002102. 10.1371/journal.pgen.1002102 21655086 PMC3107199

[23] Sellier C., Freyermuth F., Tabet R et al. Sequestration of DROSHA and DGCR8 by expanded CGG RNA repeats alters microRNA processing in fragile X-associated tremor/ataxia syndrome. Cell Rep. 2013;3:869–80. 10.1016/j.celrep.2013.02.004 23478018 PMC3639429

[24] Cid-Samper F., Gelabert-Baldrich M., Lang B et al. An integrative study of protein-RNA condensates identifies scaffolding RNAs and reveals players in fragile X-associated tremor/ataxia syndrome. Cell Rep. 2018;25:3422–34.e7. 10.1016/j.celrep.2018.11.076 30566867 PMC6315285

[25] Greco C.M., Berman R.F., Martin R.M et al. Neuropathology of fragile X-associated tremor/ataxia syndrome (FXTAS). Brain. 2006;129:243–55. 10.1093/brain/awh683 16332642

[26] Todd P.K., Oh S.Y., Krans A et al. CGG repeat associated translation mediates neurodegeneration in fragile X tremor ataxia syndrome. Neuron. 2014;78:440–55. 10.1016/j.neuron.2013.03.026 PMC3831531 23602499

[27] Kearse M.G., Green K.M., Krans A et al. CGG repeat-associated non-AUG translation utilizes a cap-dependent scanning mechanism of initiation to produce toxic proteins. Mol Cell. 2017;62:314–22. 10.1016/j.molcel.2016.02.034 PMC4854189 27041225

[28] Ariza J., Rogers H., Monterrubio A et al. A majority of FXTAS cases present with intranuclear inclusions within Purkinje cells. Cerebellum. 2016;15:546–51. 10.1007/s12311-016-0776-y 27108270

[29] Buijsen R.A., Sellier C., Severijnen L.-A.W et al. FMRpolyG-positive inclusions in CNS and non-CNS organs of a fragile X premutation carrier with fragile X-associated tremor/ataxia syndrome. Acta Neuropathol Commun. 2014;2:1–5. 10.1186/s40478-014-0162-2 25471011 PMC4254384

[30] Green K.M., Glineburg M.R., Kearse M.G et al. RAN translation at C9orf72-associated repeat expansions is selectively enhanced by the integrated stress response. Nat Commun. 2017;8:2005. 10.1038/s41467-017-02200-0 29222490 PMC5722904

[31] Wright S.E., Rodriguez C.M., Monroe J et al. CGG repeats trigger translational frameshifts that generate aggregation-prone chimeric proteins. Nucleic Acids Res. 2022;50:8674–89. 10.1093/nar/gkac626 35904811 PMC9410890

[32] Krans A., Skariah G., Zhang Y et al. Neuropathology of RAN translation proteins in fragile X-associated tremor/ataxia syndrome. Acta Neuropathol Commun. 2019;7:1–17. 10.1186/s40478-019-0782-7 PMC6821001 31665086

[33] Glineburg M.R., Todd P.K., Charlet-Berguerand N et al. Repeat-associated non-AUG (RAN) translation and other molecular mechanisms in fragile X tremor ataxia syndrome. Brain Res. 2018;1693:43–54. 10.1016/j.brainres.2018.02.006 29453961 PMC6010627

[34] Sellier C., Buijsen R.A.M., He F et al. Translation of expanded CGG repeats into FMRpolyG is pathogenic and may contribute to fragile X tremor ataxia syndrome. Neuron. 2017;93:331–47. 10.1016/j.neuron.2016.12.016 28065649 PMC5263258

[35] Krans A., Kearse M.G., Todd P.K. Repeat-associated non-AUG translation from antisense CCG repeats in fragile X tremor/ataxia syndrome. Ann Neurol. 2016;80:871–81. 10.1002/ana.24800 27761921 PMC5177492

[36] Johnstone T.G., Bazzini A.A., Giraldez A.J. Upstream ORF s are prevalent translational repressors in vertebrates. EMBO J. 2016;35:706–23. 10.15252/embj.201592759 26896445 PMC4818764

[37] Rodriguez C.M., Wright S.E., Kearse M.G et al. A native function for RAN translation and CGG repeats in regulating fragile X protein synthesis. Nat Neurosci. 2020;23:386–97. 10.1038/s41593-020-0590-1 32066985 PMC7668390

[38] Tutak K., Broniarek I., Zielezinski A et al. Ribosomal composition affects the noncanonical translation and toxicity of polyglycine-containing proteins in fragile X-associated conditions. eLife. 2024;13:RP98631. 10.7554/eLife.98631 PMC12084008 40377206

[39] Baud A., Saha D., Skrzypczak T et al. IGF2BPs directly regulate the noncanonical translation of toxic proteins from mutant FMR1 mRNA containing expanded CGG repeats. Nat Commun. 2025;17:569. 10.1038/s41467-025-67261-y 41372183 PMC12808120

[40] Konieczny P., Mukherjee S., Stepniak-Konieczna E et al. Cyclic mismatch binding ligands interact with disease-associated CGG trinucleotide repeats in RNA and suppress their translation. Nucleic Acids Res. 2021;49:9479–95. 10.1093/nar/gkab669 34358321 PMC8450082

[41] Paek K.Y., Hong K.Y., Ryu I et al. Translation initiation mediated by RNA looping. Proc Nat Acad Sci USA. 2015;112:1041–6. 10.1073/pnas.1416883112 25583496 PMC4313796

[42] Livak K.J., Schmittgen T.D. Analysis of relative gene expression data using real-time quantitative PCR and the 2-ΔΔCT method. Methods. 2001;25:402–8. 10.1006/meth.2001.1262 11846609

[43] Susorov D., Egri S., Korostelev A.A. Termi-Luc: a versatile assay to monitor full-protein release from ribosomes. RNA. 2020;26:2044–50. 10.1261/rna.076588.120 32817446 PMC7668252

[44] Napierala M., Michalowski D., de Mezer M et al. Facile FMR1 mRNA structure regulation by interruptions in CGG repeats. Nucleic Acids Res. 2005;33:451–63. 10.1093/nar/gki186 15659577 PMC548340

[45] Zhang Y., Glineburg M.R., Basrur V et al. Mechanistic convergence across initiation sites for RAN translation in fragile X associated tremor ataxia syndrome. Hum Mol Genet. 2022;31:2317–32. 10.1093/hmg/ddab353 35137065 PMC9307318

[46] De Arce A.J.D., Noderer W.L., Wang C.L. Complete motif analysis of sequence requirements for translation initiation at non-AUG start codons. Nucleic Acids Res. 2018;46:985–94. 29228265 10.1093/nar/gkx1114 PMC5778536

[47] Kozak M . Point mutations define a sequence flanking the AUG initiator codon that modulates translation by eukaryotic ribosomes. Cell. 1986;44:283–92. 10.1016/0092-8674(86)90762-2 3943125

[48] Kozak M . Compilation and analysis of sequences upstream from the translational start site in eukaryotic mRNAs. Nucleic Acids Res. 1984;12:857–72. 10.1093/nar/12.2.857 6694911 PMC318541

[49] Pisarev A.V., Kolupaeva V.G., Pisareva V.P et al. Specific functional interactions of nucleotides at key −3 and +4 positions flanking the initiation codon with components of the mammalian 48S translation initiation complex. Genes Dev. 2006;20:624–36. 10.1101/gad.1397906 16510876 PMC1410799

[50] Kozak M . Downstream secondary structure facilitates recognition of initiator codons by eukaryotic ribosomes. Proc Natl Acad Sci USA. 1990;87:8301–5. 10.1073/pnas.87.21.8301 2236042 PMC54943

[51] Tidu A., Alghoul F., Despons L et al. Critical *cis*-parameters influence STructure assisted RNA translation (START) initiation on non-AUG codons in eukaryotes. NAR Genom Bioinform. 2024;6:lqae065. 10.1093/nargab/lqae065 38863530 PMC11165317

[52] Brar Gloria A., Weissman Jonathan S.. Ribosome profiling reveals the what, when, where, and how of protein synthesis. Nat Rev Mol Cell Biol. 2015;16:651–64. 10.1038/nrm4069 26465719 PMC5522010

[53] Derbis M., Kul E., Niewiadomska D et al. Short antisense oligonucleotides alleviate the pleiotropic toxicity of RNA harboring expanded CGG repeats. Nat Commun. 2021;12:1–17. 10.1038/s41467-021-21021-w 33627639 PMC7904788

[54] Liang X.H., Shen W., Sun H et al. Translation efficiency of mRNAs is increased by antisense oligonucleotides targeting upstream open reading frames. Nat Biotechnol. 2016;34:875–80. 10.1038/nbt.3589 27398791

[55] Liang X.H., Sun H., Shen W et al. Antisense oligonucleotides targeting translation inhibitory elements in 5′ UTRs can selectively increase protein levels. Nucleic Acids Res. 2017;45:9528–46. 10.1093/nar/gkx632 28934489 PMC5766168

[56] Aspden J.L., Eyre-Walker Y.C., Phillips R.J et al. Extensive translation of small open reading frames revealed by poly-ribo-seq. eLife. 2014;3:1–19. 10.7554/eLife.03528 PMC4359375 25144939

[57] Kozak M . Influences of mRNA secondary structure on initiation by eukaryotic ribosomes. Proc Natl Acad Sci USA. 1986;83:2850–4. 10.1073/pnas.83.9.2850 3458245 PMC323404

[58] Kozak M . Point mutations close to the AUG initiator codon affect the efficiency of translation of rat preproinsulin *in vivo*. Nature. 1984;308:241–6. 10.1038/308241a0 6700727

[59] Kozak M . Context effects and inefficient initiation at non-AUG codons in eucaryotic cell-free translation systems. Mol Cell Biol. 1989;9:5073–80. 2601709 10.1128/mcb.9.11.5073-5080.1989 PMC363659

[60] Andreev D.E., Loughran G., Fedorova A.D et al. Non-AUG translation initiation in mammals. Genome Biol. 2022;23:1–17. 10.1186/s13059-022-02674-2 35534899 PMC9082881

[61] Kochetov A.V., Palyanov A., Titov I.I et al. AUG hairpin: prediction of a downstream secondary structure influencing the recognition of a translation start site. BMC Bioinf. 2007;8:3–9. 10.1186/1471-2105-8-318 PMC2001202 17760957

[62] Nadel Y., Weisman-Shomer P., Fry M. The fragile X syndrome single strand D(CGG)(n) nucleotide repeats readily fold back to form unimolecular hairpin structures. J Biol Chem. 1995;270:28970–7. 10.1074/jbc.270.48.28970 7499428

[63] Khateb S., Weisman-Shomer P., Hershco-Shani I et al. The tetraplex (CGG)n destabilizing proteins hnRNP A2 and CBF-A enhance the *in vivo* translation of fragile X premutation mRNA. Nucleic Acids Res. 2007;35:5775–88. 10.1093/nar/gkm636 17716999 PMC2034458

[64] Ciesiolka A., Jazurek M., Drazkowska K et al. Structural characteristics of simple RNA repeats associated with disease and their deleterious protein interactions. Front Cell Neurosci. 2017;11:1–19. 10.3389/fncel.2017.00097 28442996 PMC5387085

[65] Brcic J., Plavec J. NMR structure of a G-quadruplex formed by four d(G4C2) repeats: insights into structural polymorphism. Nucleic Acids Res. 2018;46:11605–17. 30277522 10.1093/nar/gky886 PMC6265483

[66] Goodman L.D., Bonini N.M. Repeat-associated non-AUG (RAN) translation mechanisms running into focus for GGGGCC-repeat associated ALS/FTD. Prog Neurobiol. 2019;183:139–48. 10.1016/j.pneurobio.2019.101697 PMC6941355 31550516

[67] Lee S., Liu B., Lee S et al. Global mapping of translation initiation sites in mammalian cells at single-nucleotide resolution. Proc Natl Acad Sci USA. 2012;109:E2424–32. 10.1073/pnas.1207846109 22927429 PMC3443142

[68] Andreev D.E., O’Connor P.B.F., Loughran G et al. Insights into the mechanisms of eukaryotic translation gained with ribosome profiling. Nucleic Acids Res. 2017;45:513–26. 10.1093/nar/gkw1190 27923997 PMC5314775

[69] Kearse M.G., Goldman D.H., Choi J et al. Ribosome queuing enables non-AUG translation to be resistant to multiple protein synthesis inhibitors. Genes Dev. 2019;33:871–85. 10.1101/gad.324715.119 31171704 PMC6601509

[70] Pelletier J., Sonenberg N. Insertion mutagenesis to increase secondary structure within the 5′ noncoding region of a eukaryotic mRNA reduces translational efficiency. Cell. 1985;40:515–26. 10.1016/0092-8674(85)90200-4 2982496

[71] Kozak M . Circumstances and mechanisms of inhibition of translation by secondary structure in eucaryotic mRNAs. Mol Cell Biol. 1989;9:5134–42. 2601712 10.1128/mcb.9.11.5134-5142.1989 PMC363665

[72] Kozak M . How do eucaryotic ribosomes select initiation regions in messenger RNA?. Cell. 1978;15:1109–23. 10.1016/0092-8674(78)90039-9 215319

[73] Kozak M . The scanning model for translation: an update. J Cell Biol. 1989;108:229–41. 10.1083/jcb.108.2.229 2645293 PMC2115416

