# Supplementary material for: The role of FMR1 mRNA structure on the efficiency of non-canonical translation of toxic polyglycine protein
Source: Nucleic Acids Res. 2026 Jun 16;54(11):gkag569. doi: 10.1093/nar/gkag569 (PMC13270972; doi:10.1093/nar/gkag569)
Supplement: gkag569_Supplemental_File [file gkag569_supplemental_file.pdf]

THE ROLE OF *FMR1* mRNA STRUCTURE ON THE EFFICIENCY OF NON-CANONICAL TRANSLATION OF TOXIC POLYGLYCINE PROTEIN

AUTHORS

Daria Niewiadomska[1], Agnieszka Piasecka[1], Anna Baud[1,2], Izabela Broniarek[1] and Krzysztof Sobczak[1,2,]*

[1] Department of Gene Expression, Institute of Molecular Biology and Biotechnology, Adam Mickiewicz University, Uniwersytetu Poznanskiego 6, 61-614 Poznan, Poland.
[2] Center for Development of Gene Therapies, Center for Advanced Technologies, Adam Mickiewicz University, Uniwersytetu Poznanskiego 10, 61-614 Poznan, Poland.

* To whom correspondence should be addressed. Tel: +48 61 829 5766; Fax: +48 61 829 5949; Email: ksobczak@amu.edu.pl

Supplementary Materials

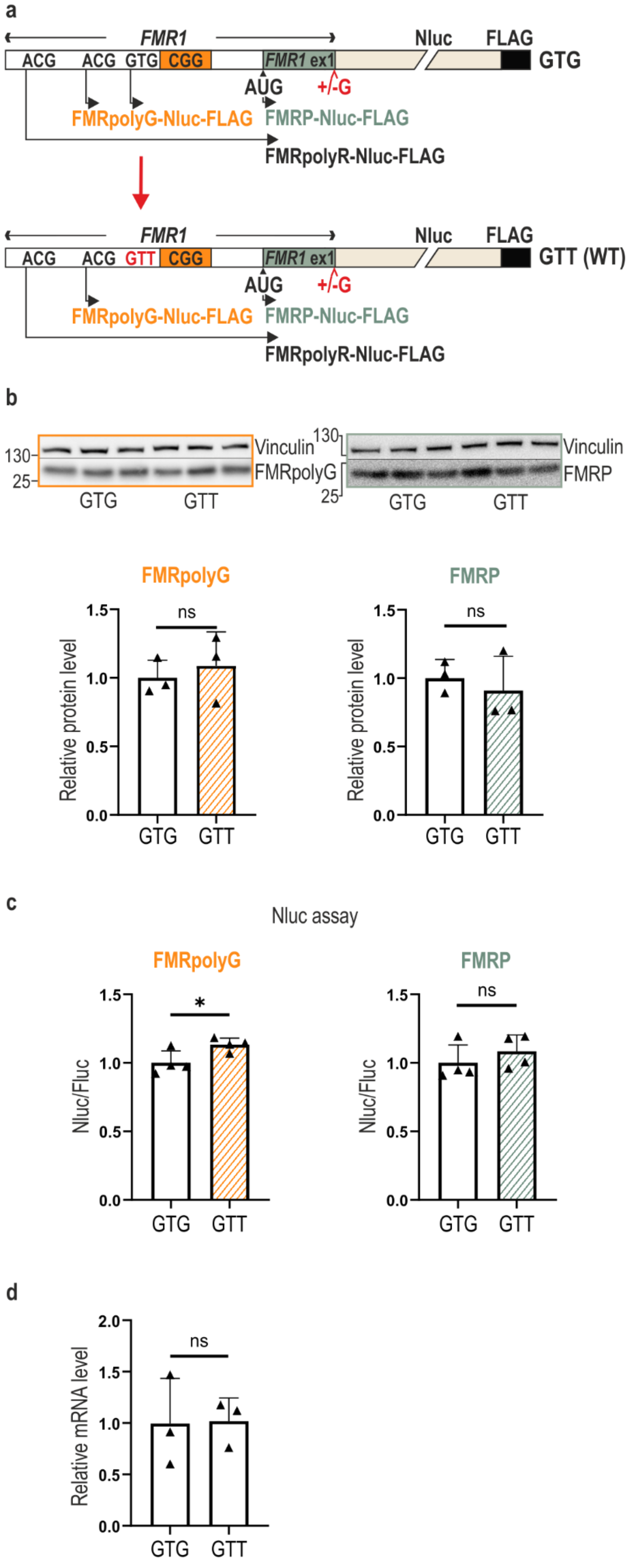
a
FMR1
Nluc
FLAG
ACG
ACG
GTG
CGG
FMR1 ex1
GTG
AUG
+/-G
FMRpolyG-Nluc-FLAG
FMRP-Nluc-FLAG
FMRpolyR-Nluc-FLAG
FMR1
Nluc
FLAG
ACG
ACG
GTT
CGG
FMR1 ex1
GTT (WT)
AUG
+/-G
FMRpolyG-Nluc-FLAG
FMRP-Nluc-FLAG
FMRpolyR-Nluc-FLAG
b
130
25
Vinculin
FMRpolyG
GTG
GTT
130
25
Vinculin
FMRP
GTG
GTT
FMRpolyG
FMRP
Relative protein level
ns
GTG
GTT
c
Nluc assay
FMRpolyG
FMRP
Nluc/Fluc
*
ns
GTG
GTT
d
Relative mRNA level
ns
GTG
GTT

Supplementary Figure S1A. Data related to Figure 1. Unification of FMRpolyG RAN translation initiation site. The GUG (+1) near-cognate start codon was mutated to ensure FMRpolyG RAN translation initiation at a single near-cognate ACG (+1) start codon.

a) Scheme of cloned constructs: GTG (upper), and GTT (lower; called WT in further analyses).

b) WB analysis and corresponding quantification of FMRpolyG-Nluc-FLAG and FMRP-Nluc-FLAG levels in HEK-293 cells 24 h post-transfection with indicated constructs. Results were normalized to Vinculin. Graphs represent averages for N = 3 biologically independent samples with SDs relative to GTG=1.

c) Quantification of results from Nluc assay. Graphs represent results for N = 4 biologically independent samples with SDs, relative to GTG=1.

d) The effect of GTG→GTT mutation on the mRNA level quantified with RT-qPCR and normalized to GAPDH. Graphs represent averages for N = 3 biologically independent samples with SDs, relative to GTG=1; Statistical analysis was performed using an unpaired two-sided t-test; *, $p<0.05$; ns, non-significant.

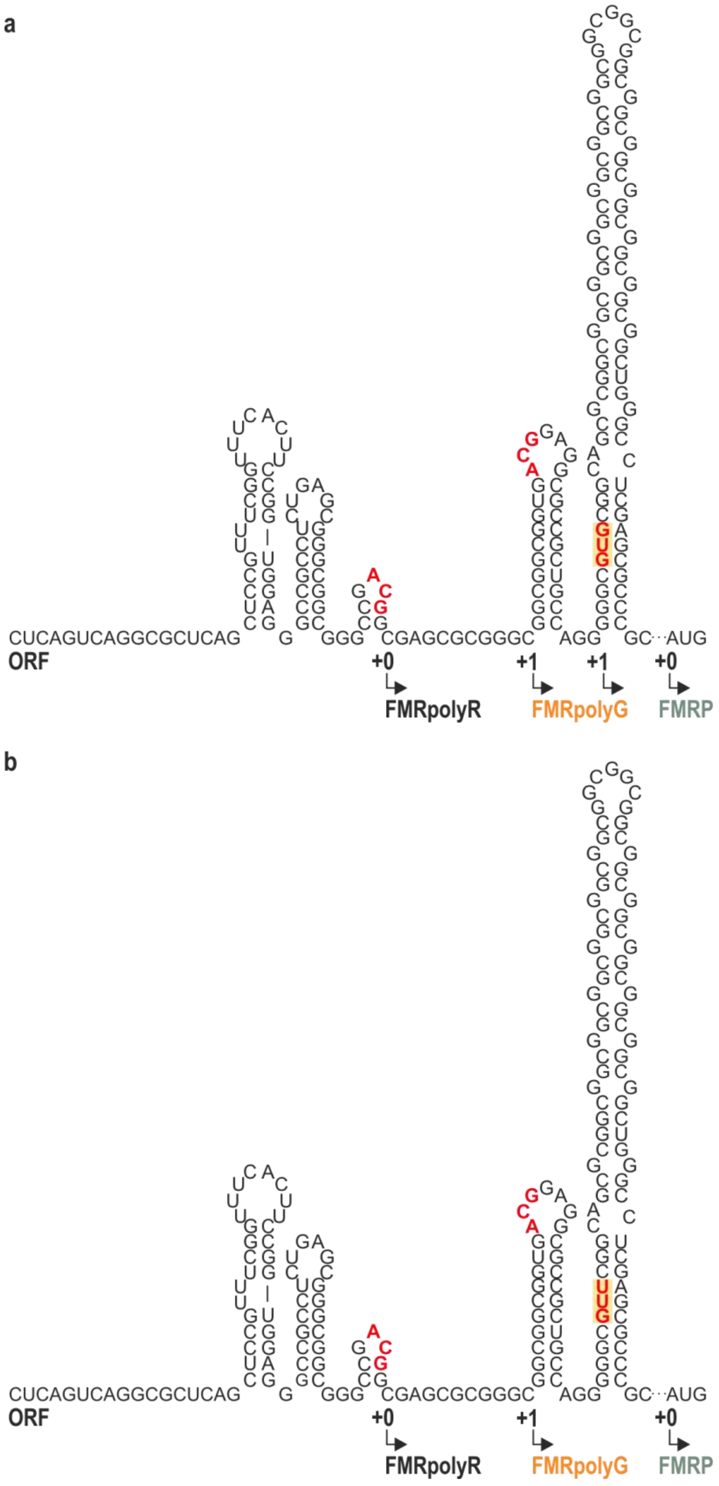

Supplementary Figure S1B. Data related to Figure 1. The predicted RNA secondary structure of *FMR1* 5′UTR highlighting the near-cognate start codons for FMRpolyR (ACG +0) and FMRpolyG (ACG +1, GUG +1) frames. The structures present the *FMR1* 5′UTR containing 16 CGGs with the **(a)** native GUG (+1) near-cognate start codon, and **(b)** GUG→GUU mutation. The mutagenesis of the GUG codon (GTG→GTT mutation) is marked in yellow.

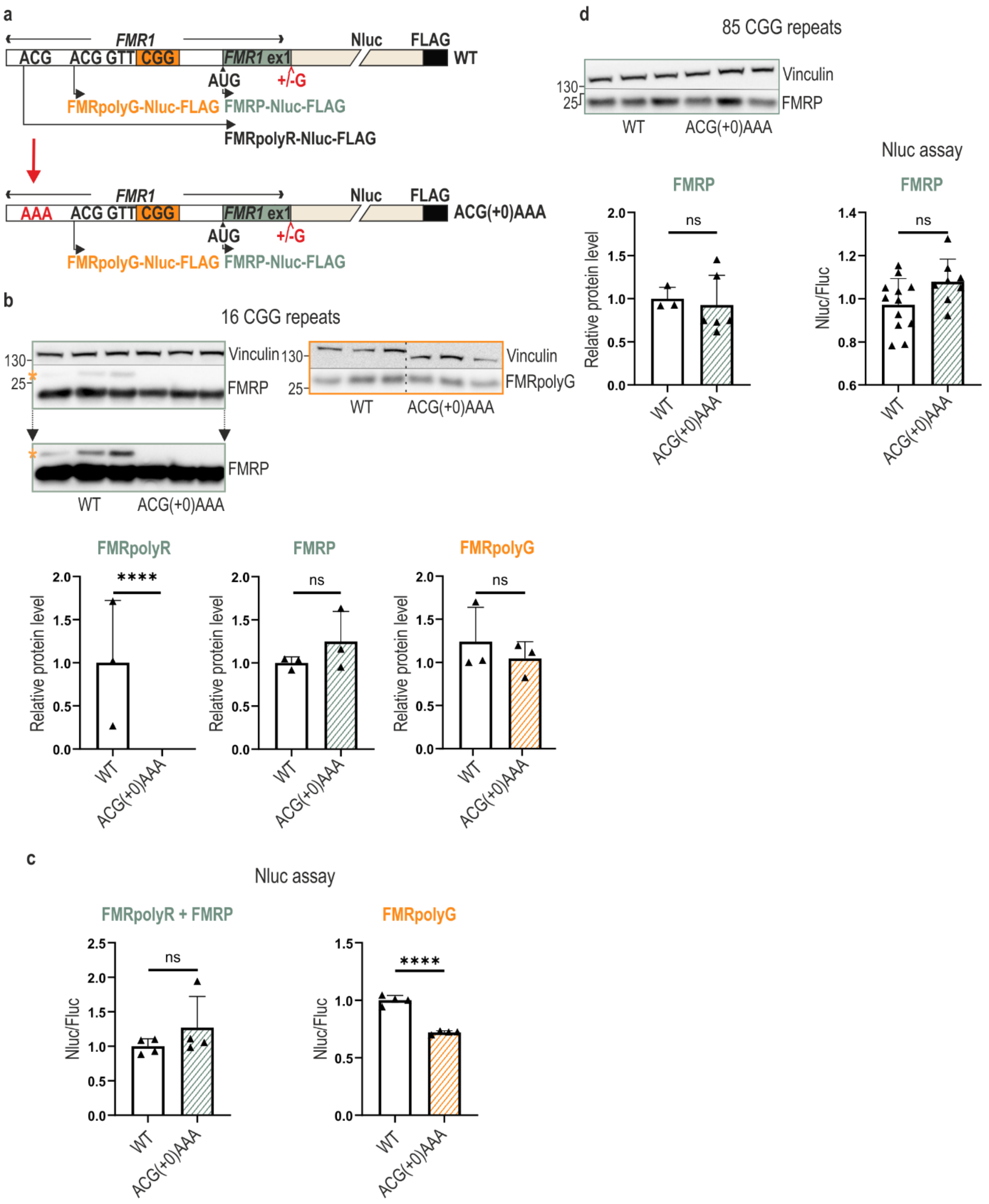

Supplementary Figure S1C. Data related to Figure 1. Mutation of ACG (+0) near-cognate start codon for FMRpolyR does not influence the level of FMRpolyG.

a) Scheme of constructs cloned to confirm that the FMRpolyR is produced from ACG (+0) near-cognate start codon; WT (upper), and ACG(+0)AAA (lower).

b) 16 CGGs: WB analysis and corresponding quantification of FMRpolyR-Nluc-FLAG, FMRP-Nluc-FLAG and FMRpolyG-Nluc-FLAG levels. Results were normalized to Vinculin. Graphs represent averages for N = 3 biologically independent samples with SDs, relative to WT=1. The FMRpolyR-Nluc-FLAG produced from FMRP-Nluc-FLAG is marked by an orange asterisk.

c) 16 CGGs: Quantification of results from Nluc assay. For the +0 frame assay detects both, FMRP-Nluc-FLAG and FMRpolyR-Nluc-FLAG proteins; however, due to the dominant level of FMRP-Nluc-FLAG protein the amount of FMRpolyR-Nluc-FLAG is omitted in the analysis. Graphs represent averages for N = 4 biologically independent samples with SDs, relative to WT=1.

d) 85 CGGs: WB analysis and corresponding quantification (bottom left) of FMRP-Nluc-FLAG level analyzed as in **b**. Results were normalized to Vinculin. Graphs represent averages for N = 3 biologically independent samples with SDs, relative to WT=1. Quantification of results from Nluc assay (bottom right). Graphs represent results for N = 12 and N = 8 biologically independent samples with SDs, relative to WT=1, for WT and ACG(+0)AAA, respectively. Statistical analysis was performed using an unpaired two-sided t-test; ****, $p<0.0001$; ns, non-significant.

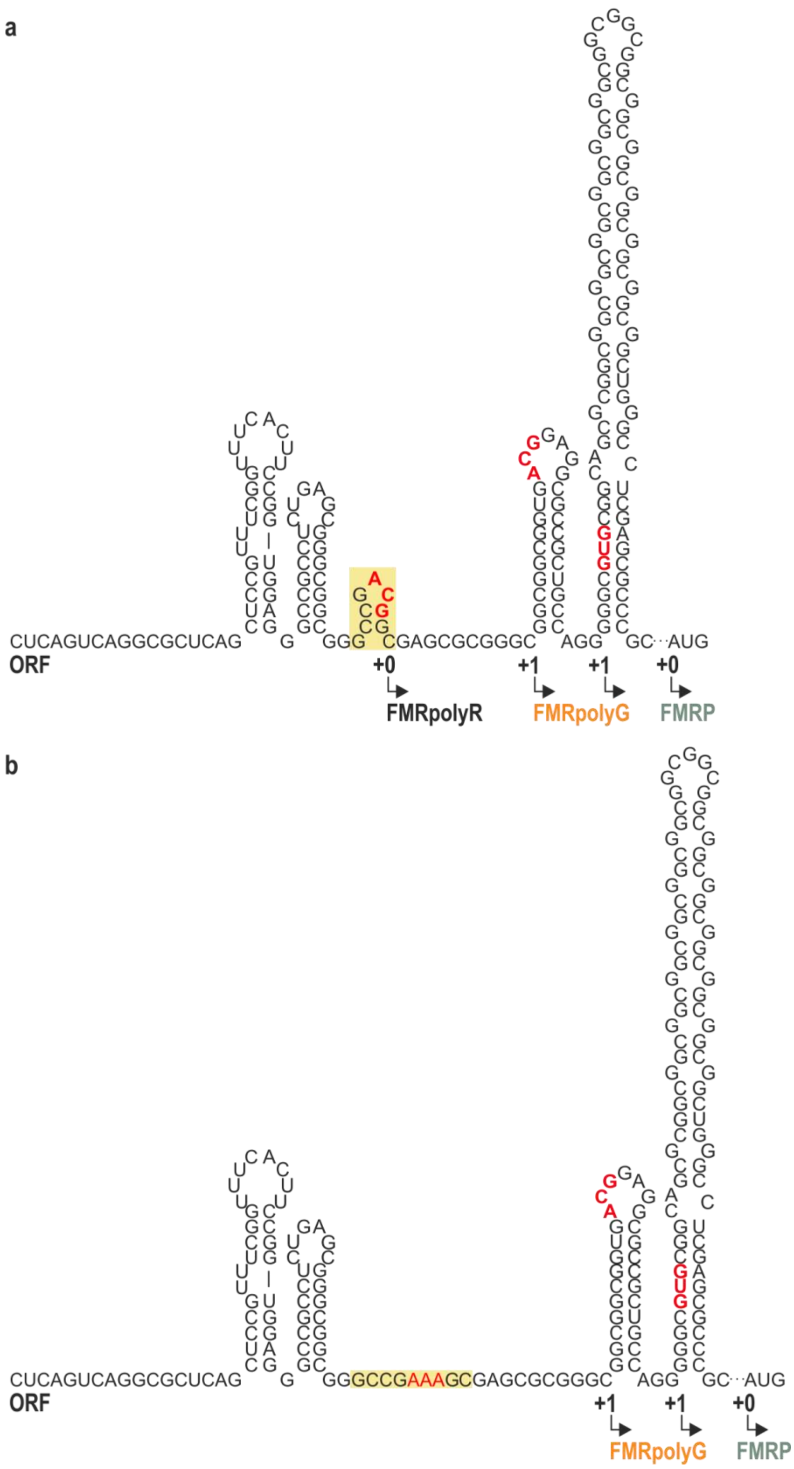

Supplementary Figure S1D. Data related to Figure 1. The predicted RNA secondary structures of *FMR1* 5′UTR containing 16 CGGs with **(a)** the native ACG (+0) near-cognate start codon, and **(b)** ACG(+0)AAA mutation. The region of the ACG (+0) codon mutagenesis is marked in yellow.

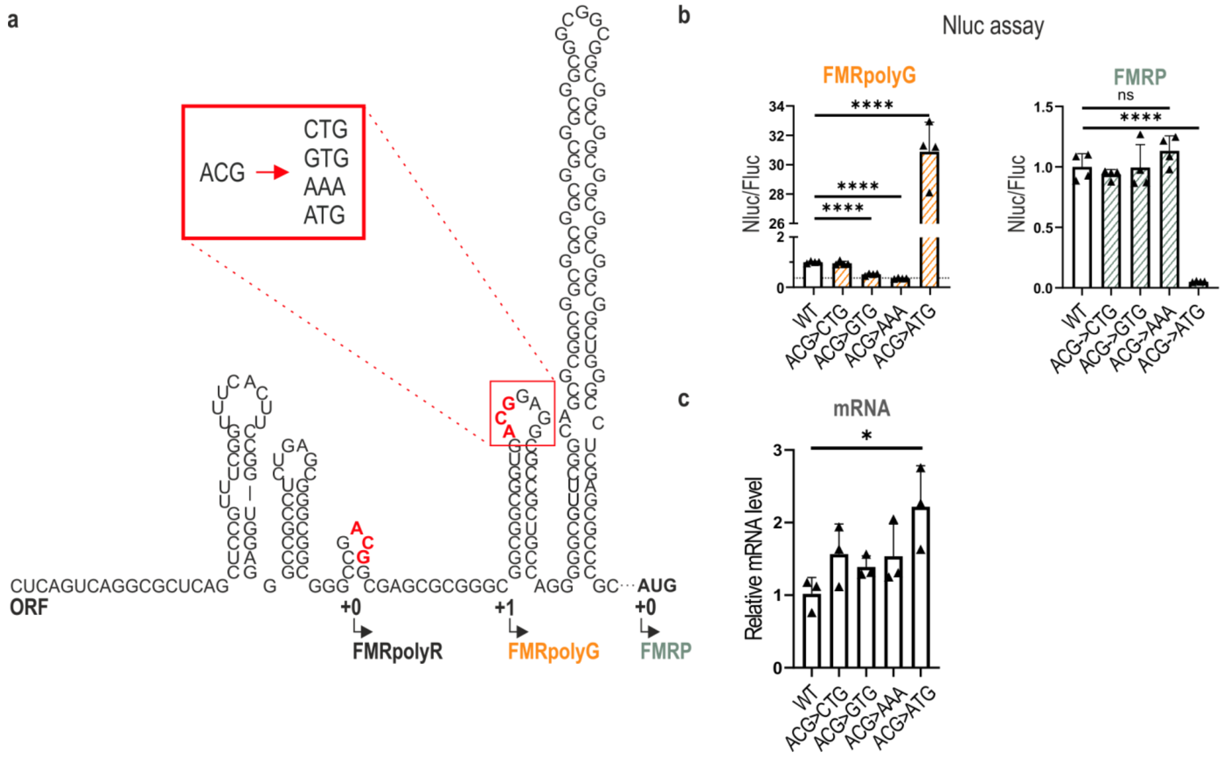

Supplementary Figure S2. Data related to Figure 2.

a) Related to Fig. 2A. The predicted secondary RNA structure of *FMR1* 5′UTR containing 16 CGGs with ACG (+1) near-cognate start codon mutation (ACG→CTG, ACG→GTG, ACG→AAA, and ACG→ATG mutations.) The region of the ACG (+1) codon mutagenesis is shown in red box.

b) Related to Fig. 2B. Quantification of results from Nluc assay. Graphs represent results for N = 4 biologically independent samples with SDs, relative to WT=1. The depth of observed changes, here and in further experiments, differ in WB and Nluc assay analyses as Nluc assay may detect all synthesized proteins that are in frame with Nluc (initiating from other near-cognate start codons). At the same time, WB is limited to the range of proteins with molecular weights of studied proteins. Those differences are particularly significant when the Nluc signal is low.

c) The effect of introduced mutations on the mRNA level quantified with RT-qPCR and normalized to GAPDH. Graphs represent averages for N = 3 biologically independent samples with SDs, relative to WT=1; Statistical analysis was performed using multiple unpaired parametric t-tests with Holm-Šídák correction for multiple comparisons; *, $p<0.05$; ****, $p<0.0001$; ns, non-significant.

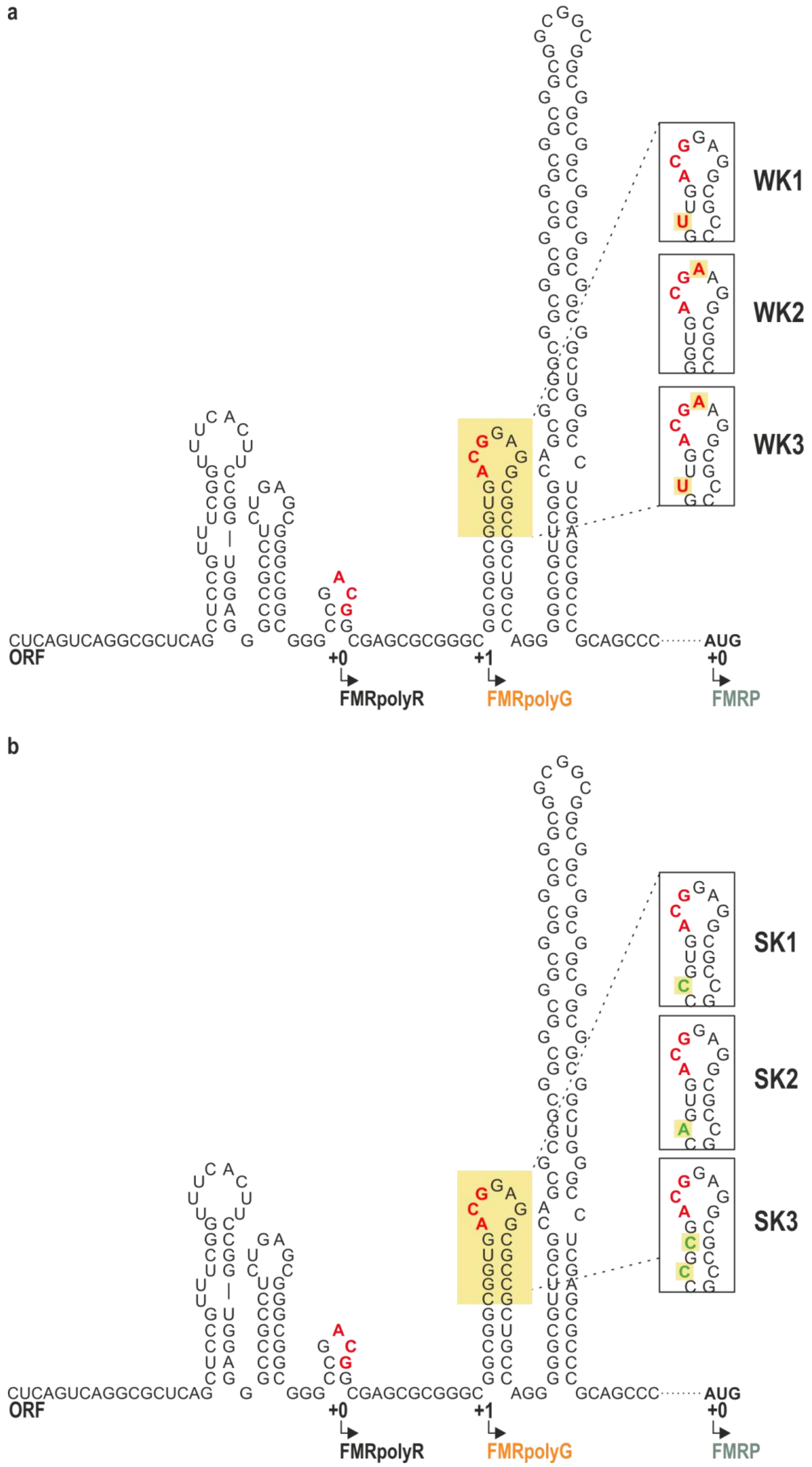

Supplementary Figure S3A. Data related to Figure 3. The predicted RNA secondary structures of *FMR1* 5'UTR containing 16 CGGs with the **a)** WK1, WK2, and WK3 mutations, and **b)** SK1, SK2, and SK3 mutations. The regions of the mutagenesis are marked in yellow.

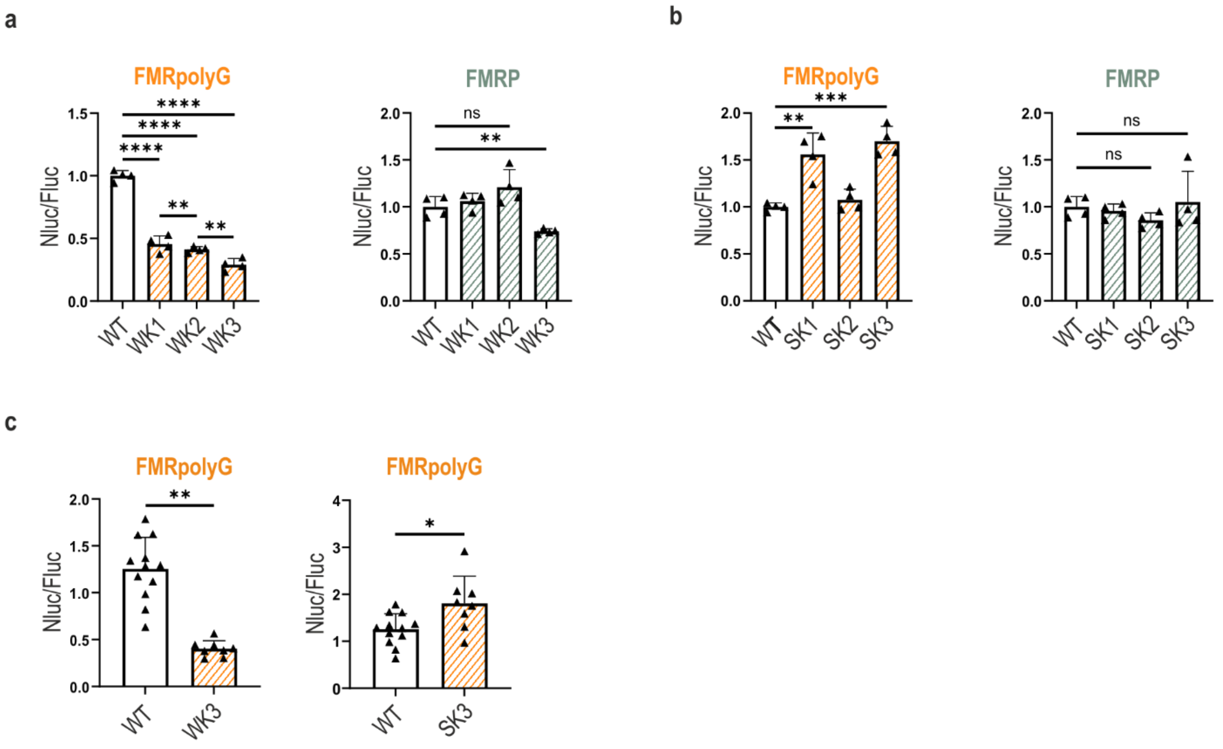

Supplementary Figure S3B. Data related to Figure 3.

a) Related to Fig. 3C. 16 CGGs: Quantification of results from Nluc assay for WK1-3 mutants. Graphs represent results for N = 4 biologically independent samples with SDs, relative to WT=1.

b) Related to Fig. 3D. 16 CGGs: Quantification of results from Nluc assay for SK1-3 mutants. Graphs represent results for N = 4 biologically independent samples with SDs, relative to WT=1.

c) Related to Fig. 3E. 85 CGGs: Quantification of results from Nluc assay for WT and WK3 (left) and WT and SK3 (right) mutants. Graphs represent results for N = 12 and N = 8 biologically independent samples with SDs, relative to WT=1, for WT and WK3 or SK3, respectively; Statistical analysis was performed using multiple unpaired parametric t-tests with Holm-Šídák correction for multiple comparisons; *, p<0.05; **, p<0.01; ***, p<0.001; ****, p<0.0001; ns, non-significant.

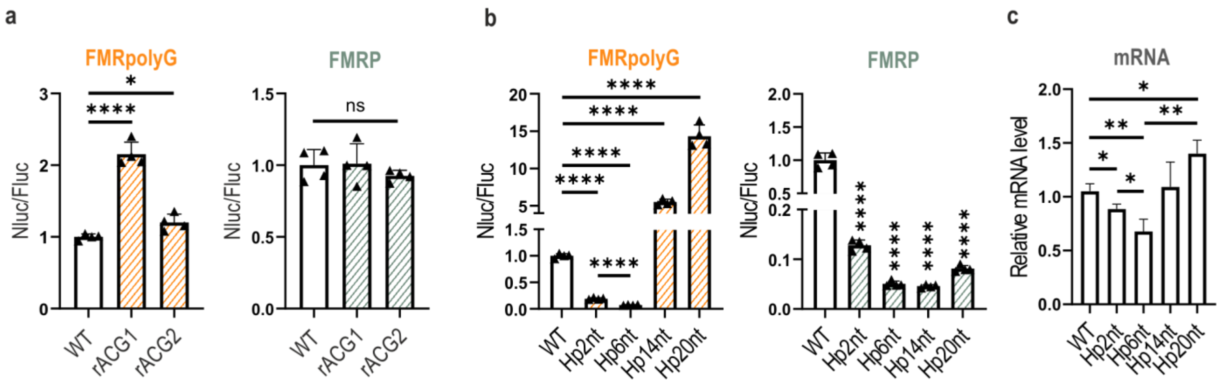

Supplementary Figure S4A. Data related to Figure 4.

a) Related to Fig. 4C. Quantification of results from Nluc assay for rACG1 and rACG2 mutants. Graphs represent results for N = 4 biologically independent samples with SDs, relative to WT=1.

b) Related to Fig. 4E. Quantification of results from Nluc assay for Hp mutants. Graphs represent results for N = 4 biologically independent samples with SDs, relative to WT=1.

c) Related to Fig. 4E. The effect of introduced mutations on the mRNA level quantified with RT-qPCR and normalized to GAPDH. Graphs represent averages for N = 3 biologically independent samples with SDs, relative to WT=1; Statistical analysis was performed using multiple unpaired parametric t-tests with Holm-Šídák correction for multiple comparisons; *, $p<0.05$; **, $p<0.01$; ****, $p<0.0001$, ns, non-significant.

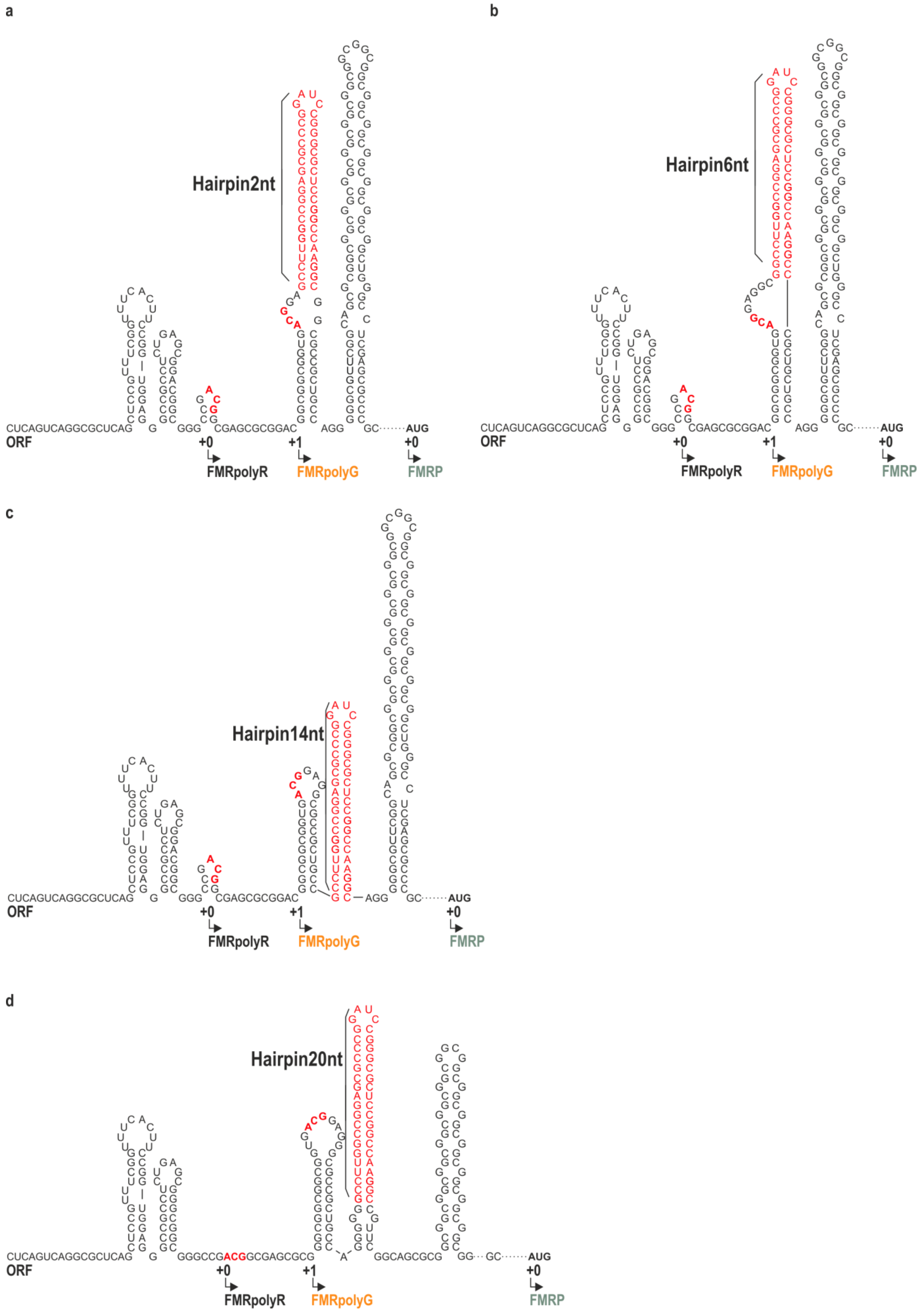

Supplementary Figure S4B. Data related to Figure 4. The predicted RNA secondary structures of *FMR1* 5′UTR containing 16 CGGs and the artificial hairpin-forming sequence

cloned: **(a)** 2 nt downstream ACG (+1) near-cognate start codon – Hp2nt, **(b)** 6 nt downstream ACG (+1) near-cognate start codon – Hp6nt, **(c)** 14 nt downstream ACG (+1) near-cognate start codon – Hp14nt, and **(d)** 20 nt downstream ACG (+1) near-cognate start codon – Hp20nt. The artificial RNA hairpin structure formed by cloned sequence is marked in red.

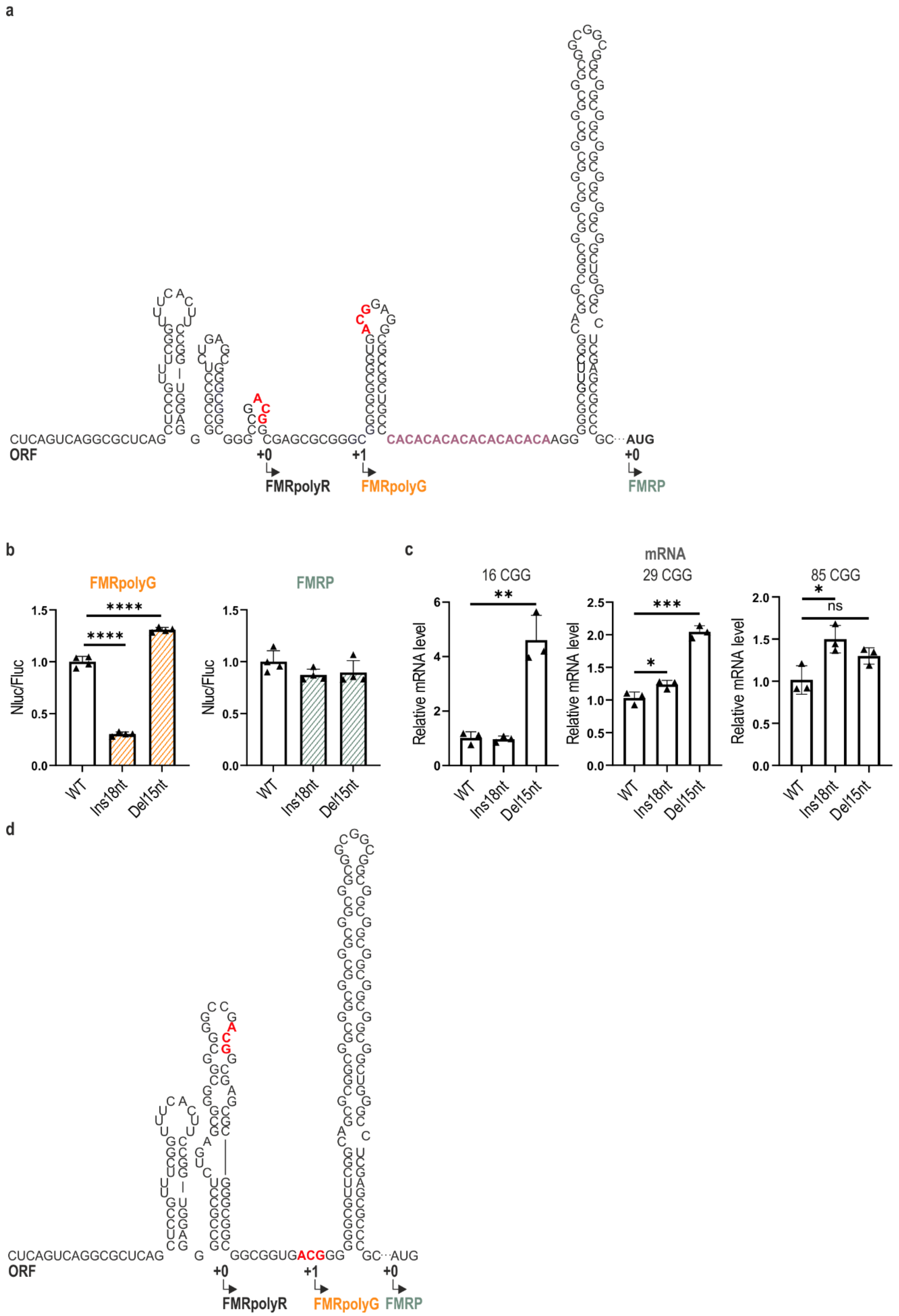

Supplementary Figure S5. Data related to Figure 5.

a) Related to Fig. 5A. The predicted RNA secondary structure of *FMR1* 5′UTR containing 16 CGGs with the Ins18nt mutation.

b) Related to Fig. 5B. 16 CGGs: Quantification of results from Nluc assay for Ins18nt and Del15nt mutants. Graphs represent results for N = 4 biologically independent samples with SDs, relative to WT=1.

c) Related to Fig. 5B–D. The effect of introduced mutations on the mRNA level quantified with RT-qPCR and normalized to GAPDH. Graphs represent results for N = 3 biologically independent samples with SDs, relative to WT=1.

d) Related to Fig. 5A. The predicted RNA secondary structure of *FMR1* 5′UTR containing 16 CGGs with the Del15nt mutation; Statistical analysis was performed using multiple unpaired parametric t-tests with Holm-Šídák correction for multiple comparisons; **, $p<0.01$; ***, $p<0.001$; ****, $p<0.0001$, ns, non-significant.

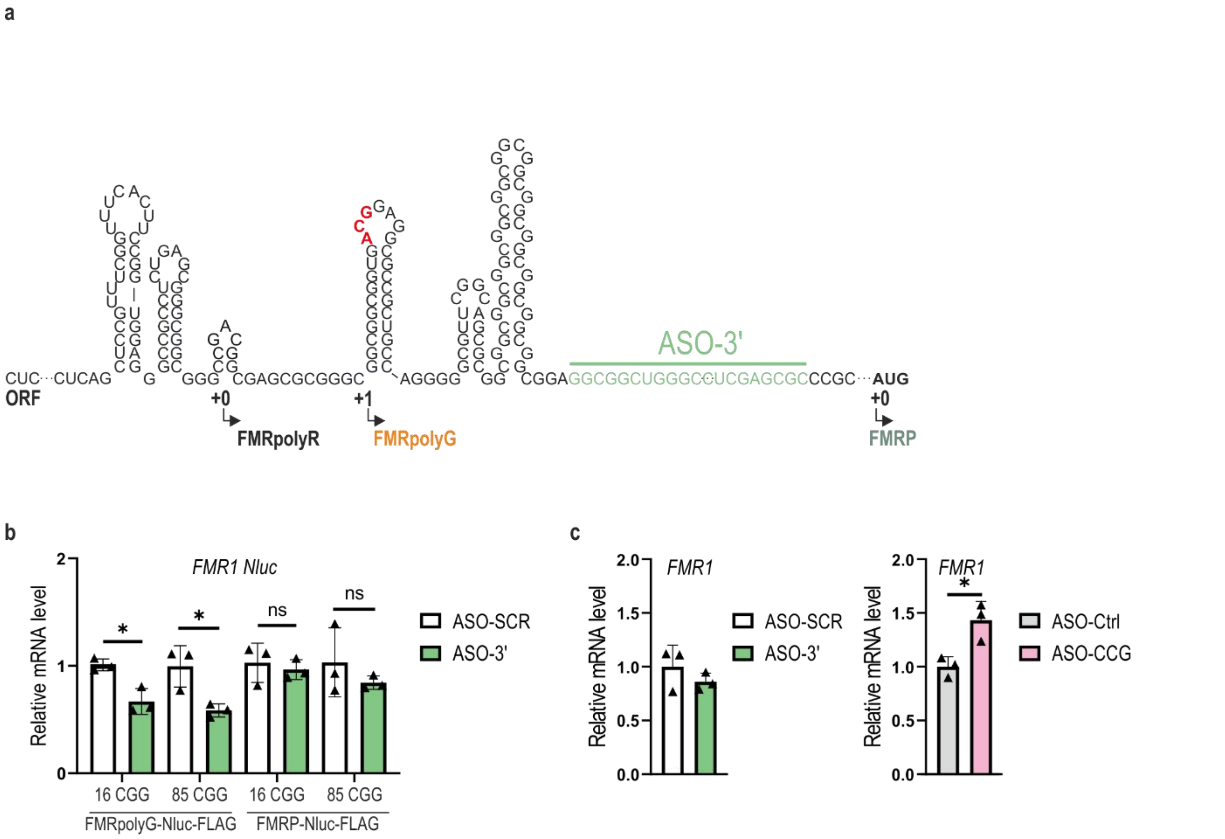

Supplementary Figure S6. Data related to Figure 6.

a) Proposed model of structural change of *FMR1* 5'UTR due to ASO-3' binding. The scheme presents the 5'UTR sequence with 16 CGG repeats.

b) Related to Fig. 6B-C. The effect of ASO administration on the mRNA level quantified with RT-qPCR and normalized to GAPDH. Graphs represent results for N = 3 biologically independent samples with SDs, relative to 16 CGG FMRpolyG-Nluc-FLAG+ASO-SCR/FMRP-Nluc-FLAG+ASO-SCR=1.

c) Related to Fig. 6E. The effect of ASO administration on the *FMR1* expression in FXTAS iPSC-derived neurons quantified with RT-qPCR and normalized to GAPDH. Graphs represent results for N = 3 biologically independent samples with SDs, relative to ASO-SCR=1 (left) and ASO-Ctrl=1 (right). Statistical analysis was performed using an unpaired two-sided t-test; *, $p<0.05$, ns, non-significant.

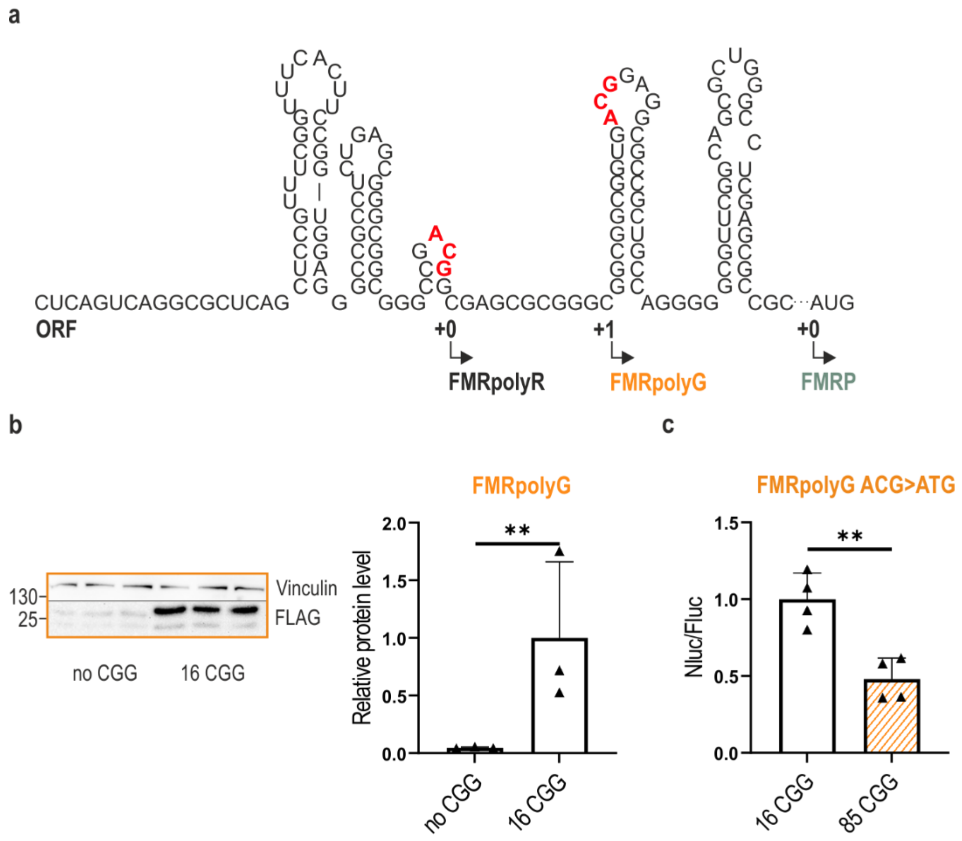

Supplementary Figure S7. Data related to Figure 7.

a) Related to Fig. 7A. The predicted RNA secondary structure of *FMR1* 5'UTR without CGG repeats.

b) Related to Fig. 7B. WB analysis and corresponding quantification of FMRpolyG-Nluc-FLAG level in HEK-293 cells 24 h post-transfection with construct without CGG repeats. The RAN product was detected via anti-FLAG antibody. Graph represents averages for N = 3 biologically independent samples with SDs, relative to 16 CGG=1.

c) Related to Fig. 7F. Quantification of results from Nluc assay for ACG>ATG constructs with 16 and 85 CGGs. Graph represents results for N = 4 biologically independent samples with SDs, relative to 16 CGG=1; Statistical analysis was performed using an unpaired two-sided t-test; **, $p<0.01$.

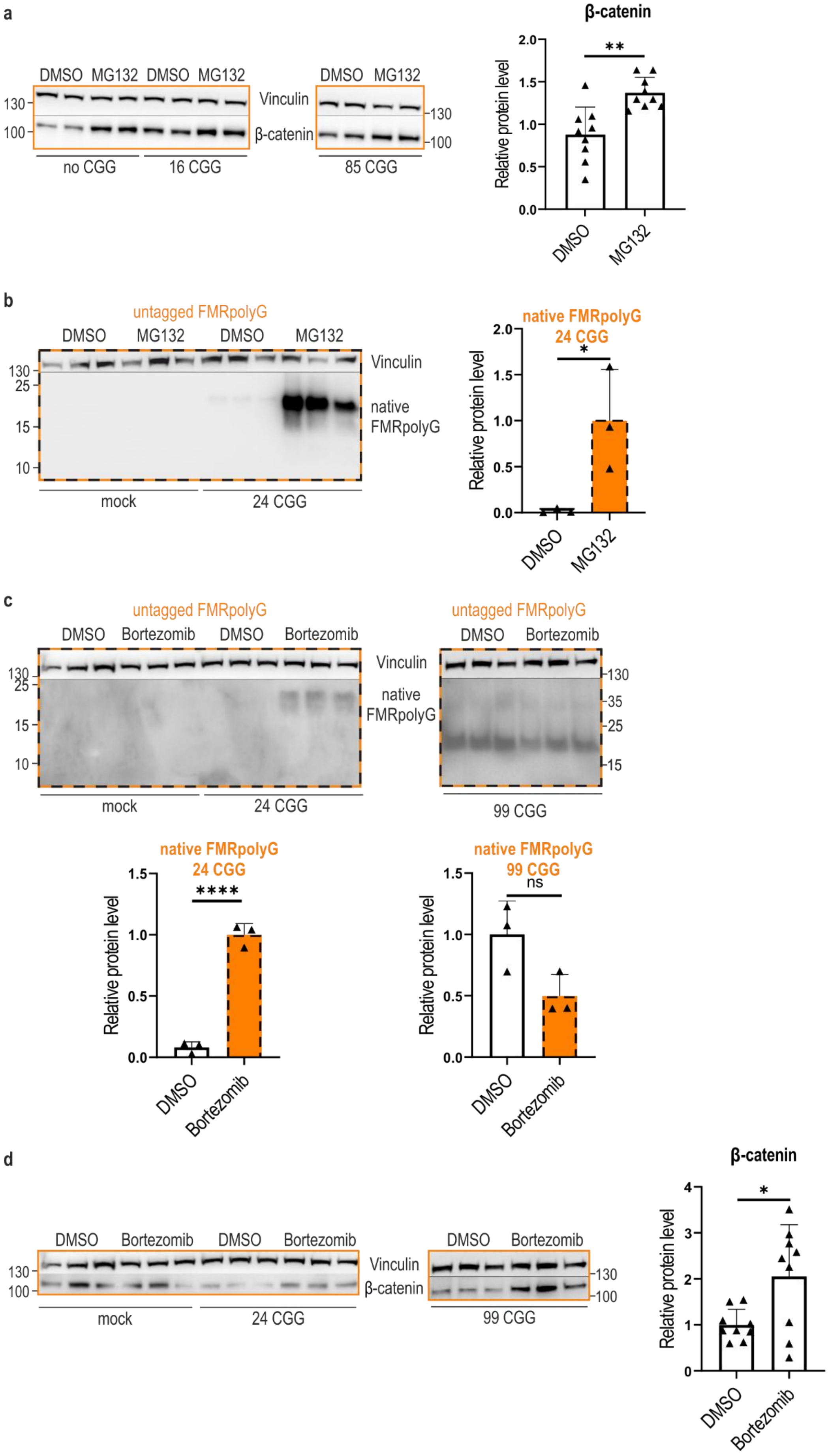
a
β-catenin
DMSO MG132 DMSO MG132
DMSO MG132
130
100
Vinculin
β-catenin
130
100
no CGG
16 CGG
85 CGG
**
Relative protein level
2.0
1.5
1.0
0.5
0.0
DMSO
MG132
b
untagged FMRpolyG
DMSO MG132 DMSO MG132
130
25
15
10
Vinculin
native FMRpolyG
mock
24 CGG
native FMRpolyG 24 CGG
*
Relative protein level
2.0
1.5
1.0
0.5
0.0
DMSO
MG132
c
untagged FMRpolyG
DMSO Bortezomib DMSO Bortezomib
130
25
15
10
Vinculin
native FMRpolyG
mock
24 CGG
untagged FMRpolyG
DMSO Bortezomib
130
35
25
15
99 CGG
native FMRpolyG 24 CGG
****
Relative protein level
1.5
1.0
0.5
0.0
DMSO
Bortezomib
native FMRpolyG 99 CGG
ns
Relative protein level
1.5
1.0
0.5
0.0
DMSO
Bortezomib
d
β-catenin
DMSO Bortezomib DMSO Bortezomib
130
100
Vinculin
β-catenin
mock
24 CGG
DMSO Bortezomib
130
100
99 CGG
*
Relative protein level
4
3
2
1
0
DMSO
Bortezomib

Supplementary Figure S8. Data related to Figure 8.

a) WB analysis and corresponding quantification of β-catenin level in HEK-293 cells transfected with WT constructs in +1 frame containing different number of CGGs followed by 5 μM MG132 or DMSO control treatment for 4 h. The β-catenin was used as positive control of proteasome inhibition. Graph represents averages for N = 3 biologically independent samples with SDs, relative to DMSO=1.

b) WB analysis and corresponding quantification of native, untagged FMRpolyG levels in mock-treated HEK-293 cells and cells transfected with a construct encoding *FMR1* 5'UTR containing 24 CGG repeats in the +1 frame without any tag, followed by treatment with 5 μM MG132 or DMSO control treatment for 4 h. Graph represents averages for N = 3 biologically independent samples with SDs, relative to MG132=1.

c) WB analysis and corresponding quantification of native, untagged FMRpolyG level in mock-treated HEK-293 cells and cells transfected with constructs encoding *FMR1* 5'UTR in the +1 frame without any tag, followed by treatment with 200 nM Bortezomib or DMSO control treatment for 4 h. Graph represents averages for N = 3 biologically independent samples with SDs, relative to Bortezomib=1 (left panel) and DMSO=1 (right panel).

d) WB analysis and corresponding quantification of β-catenin level in mock-treated HEK-293 cells and cells transfected with WT constructs in +1 frame containing different number of CGGs followed by 200 nM Bortezomib or DMSO control treatment for 4 h. The β-catenin was used as positive control of proteasome inhibition. Graph represents averages for N = 3 biologically independent samples with SDs, relative to DMSO=1. Results in a–d were normalized to Vinculin; Statistical analysis was performed using an unpaired two-sided t-test; *, $p<0.05$; **, $p<0.01$; ****, $p<0.0001$, ns, non-significant.

Supplementary Table1. List of oligonucleotides.

Name	Sequence
F1	TTACAAGGATGACGACGATAAGTAATTCTAGAGTCGGGGCGGC
R1	TCGTCATCCTTGTAATCTCCCGCCAGAATGCGTTCGCAC
F2	ATGGTCTTCACACTCGAAGATTTCG
R2	ACGGTTCACTAAACGAGC
F3	CGTTTAGTGAACCGTCTCAGTCAGGCGCTCAGC
R3	GAGTGTGAAGACCATCTTGTAGAAAGCGCCATTGGAGC
F4	CGTTTAGTGAACCGTCTCAGTCAGGCGCTCAGCT
R4	GAGTGTGAAGACCATCCTTGTAGAAAGCGCCATTGGA
F5	AGCGCTTTCTACAAGGGGGGTCTTCACACTCGAAGATTTCGTTG
R5	CTTGTAGAAAGCGCTTTTGGAGCCCCGCACTTCCAC
F6	CGGCGGCGTTGACGGAGGCGCCGCTGC
R6	CCGTCAACGCCGCCGCCCGCGCTCG
F7	CGGTGACGAAGGCGCCGCTGCCAGGGG
R7	GCGCCTTCGTCACCGCCGCCGCCCG
F8	CGGCGTTGACGAAGGCGCCGCTGCCAGGGG
R8	CCTTCGTCAACGCCGCCGCCCGCGCTCG
F9	GCGGCGGCCGTGACGGAGGCGCCGCTGC
R9	CGTCACGGCCGCCGCCCGCGCTCGC
F10	GCGGCGGCAGTGACGGAGGCGCCGCTGC
R10	CGTCACTGCCGCCGCCCGCGCTCGC
F11	CGGCGGCCGCGACGGAGGCGCCGCTGCC
R11	CCGTCGCGGCCGCCGCCCGCGCTCGC
F12	GGCGGTGCTGGAGGCGCCGCTGCCAGGG
R12	GCCTCCAGCACCGCCGCCGCCCGCGC
F13	GGCGGTGGTGGAGGCGCCGCTGCCAGGG
R13	GCCTCCACCACCGCCGCCGCCCGCGC
F14	GCGGTGAAAGAGGCGCCGCTGCCAGGG
R14	CGCCTCTTTCACCGCCGCCGCCCGCG
F15	CTGAGCGGACGGCGGGCCGACGGCGAG
R15	CCGCCGTCCGCTCAGAGGCGGCCCTC
F16	CGCGGGCGACGGCGGTGACGGAGGCGC
R16	CCGCCGTCGCCCGCGCTCGCCGTCG
F17	CGCCCGGATCCGGGCGCTCCGGCCAAGGCGGCGCCGCTGCCAGGGGG
R17	GCCCGGATCCGGGCGCTCCGGCCAAGGCTCCGTCACCGCCGCCGCC
F18	CGCCCGGATCCGGGCGCTCCGGCCAAGGCCCGCTGCCAGGGGGCGTTCG
R18	GCCCGGATCCGGGCGCTCCGGCCAAGGCCGCCTCCGTCACCGCCGC
F19	CGCCCGGATCCGGGCGCTCCGGCCAAGGCAGGGGGCGTTCGGCAGCG
R19	GCCCGGATCCGGGCGCTCCGGCCAAGGCGGCAGCGGCGCCTCCGTCACC
F20	CGCCCGGATCCGGGCGCTCCGGCCAAGGCCGTTCGGCAGCGCGGCGG
R20	GCCCGGATCCGGGCGCTCCGGCCAAGGCCCCCCTGGCAGCGGCGCC
F21	ACACACACACACACACAAGGGGGCGTTCGGCAGCG
R21	TGTGTGTGTGTGTGTGGGCAGCGGCGCCTCCGTCACC
F22	CAGGGGGCGTTCGGCAGCGCG
R22	GCAGCGGCGCCTCCGTCAC
F23	GCGGCGGTGATGGAGGCGCCG
R23	CGCCCGCGCTCGCCGTCG
F24	GGGGCGTTCGGCAG

R24	CCGTCACCGCCGCC
F25	CTGGGCCTCGAGCG
R25	CGCTGCCGAACGCC
F26	CGGCGGGCCGAAAGCGAGCGCGG
R26	CCCGCTCAGAGGCGGCCC
F27	CAGGGGGCGTTCGCGAGCGCGGCGG
R27	GCAGCGGCGCCTCCGTCA
F28	GGGGCGTTCGCGAGCGCGGCGG
R28	CTGGCAGCGGCGCCTCCG
F29	CATCAAGTGTATCATATGCCAAGTCC
R29	CCGCGCTCGCGAACG
F30	TAATACGACTCACTATAGGGAGAGTCTCAGTCAGGCGCTCAG
R30	CACCTGTCCTACGAGTTGCA
F31	CGCTCAGCTCCGTTTCG
R31	CACCAGCTCCTCCATCTTCT
R32	CTAGCGCCGGGAGCCCGCCCCC
F_hGAPDH_mRNA	GAGTCAACGGATTTGGTCGT
R_hGAPDH_mRNA	TTGATTTTGGAGGGATCTCG
F_FMR1_Nluc_mRNA	GCAGGGCTGAAGAGAAGATG
R_FMR1_Nluc_mRNA	TGGATCGGAGTTACGGACAC
